# Effects of tofersen treatment in patients with *SOD1*-ALS in a “real-world” setting – a 12-month multicenter cohort study from the German early access program

**DOI:** 10.1016/j.eclinm.2024.102495

**Published:** 2024-02-15

**Authors:** Maximilian Wiesenfarth, Johannes Dorst, David Brenner, Zeynep Elmas, Özlem Parlak, Zeljko Uzelac, Katharina Kandler, Kristina Mayer, Ulrike Weiland, Christine Herrmann, Joachim Schuster, Axel Freischmidt, Kathrin Müller, Reiner Siebert, Franziska Bachhuber, Tatiana Simak, Kornelia Günther, Elke Fröhlich, Antje Knehr, Martin Regensburger, Alexander German, Susanne Petri, Julian Grosskreutz, Thomas Klopstock, Peter Reilich, Florian Schöberl, Tim Hagenacker, Ute Weyen, René Günther, Maximilian Vidovic, Martin Jentsch, Thomas Haarmeier, Patrick Weydt, Ivan Valkadinov, Jasper Hesebeck-Brinckmann, Julian Conrad, Jochen Hans Weishaupt, Peggy Schumann, Peter Körtvélyessy, Thomas Meyer, Wolfgang Philipp Ruf, Simon Witzel, Makbule Senel, Hayrettin Tumani, Albert Christian Ludolph

**Affiliations:** a Department of Neurology, Ulm University, 89081, Ulm, Germany; b German Centre for Neurodegenerative Diseases (DZNE) Site Ulm, 89081, Ulm, Germany; c Institute of Human Genetics, Ulm University and Ulm University Medical Center, 89081, Ulm, Germany; d Department of Molecular Neurology, Friedrich-Alexander-Universität Erlangen-Nürnberg (FAU), 91054, Erlangen, Germany; e Deutsches Zentrum Immuntherapie (DZI), University Hospital Erlangen, 91054, Erlangen, Germany; f Department of Neurology, Hannover Medical School, 30625, Hannover, Germany; g Precision Neurology of Neuromuscular and Motoneuron Diseases, University of Lübeck, 23538, Lübeck, Germany; h Department of Neurology with Friedrich-Baur-Institute, LMU University Hospital, LMU Munich, 80336, München, Germany; i German Centre for Neurodegenerative Diseases (DZNE) Site Munich, 81377, Munich, Germany; j Munich Cluster for Systems Neurology (SyNergy), 81377, Munich, Germany; k Department of Neurology and Center for Translational Neuro and Behavioral Sciences (C-TNBS), University Hospital Essen, 45127, Essen, Germany; l Department of Neurology, Ruhr-University Bochum, BG-Kliniken Bergmannsheil, 44789, Bochum, Germany; m Department of Neurology, University Hospital Carl Gustav Carus, Technische Universität Dresden, 01307, Dresden, Germany; n German Center for Neurodegenerative Diseases (DZNE) Site Dresden, 01307, Dresden, Germany; o Department of Neurology, Helios Klinikum Krefeld, 47805, Krefeld, Germany; p Department for Neurodegenerative Disorders and Gerontopsychiatry, Bonn University, 53127, Bonn, Germany; q German Centre for Neurodegenerative Diseases (DZNE) Site Bonn, 53127, Bonn, Germany; r Division for Neurodegenerative Diseases, Neurology Department, Mannheim Center for Translational Medicine, University Medicine Mannheim, Heidelberg University, 68167, Mannheim, Germany; s Ambulanzpartner Soziotechnologie GmbH, 13353, Berlin, Germany; t Department of Neurology, Center for ALS and Other Motor Neuron Disorders, Charité - Universitätsmedizin Berlin, Corporate Member of Freie Universität Berlin, Humboldt-Universität zu Berlin, and Berlin Institute of Health, 13353, Berlin, Germany; u German Centre for Neurodegenerative Diseases (DZNE) Site Magdeburg, 39120, Magdeburg, Germany

**Keywords:** Amyotrophic lateral sclerosis, *SOD1*, Antisense oligonucleotide, Tofersen, Early access program

## Abstract

**Background:**

In April 2023, the antisense oligonucleotide tofersen was approved by the U.S. Food and Drug Administration (FDA) for treatment of *SOD1*-amyotrophic lateral sclerosis (ALS), after a decrease of neurofilament light chain (NfL) levels had been demonstrated.

**Methods:**

Between 03/2022 and 04/2023, 24 patients with *SOD1*-ALS from ten German ALS reference centers were followed-up until the cut-off date for ALS functional rating scale revised (ALSFRS-R), progression rate (loss of ALSFRS-R/month), NfL, phosphorylated neurofilament heavy chain (pNfH) in cerebrospinal fluid (CSF), and adverse events.

**Findings:**

During the observation period, median ALSFRS-R decreased from 38.0 (IQR 32.0–42.0) to 35.0 (IQR 29.0–42.0), corresponding to a median progression rate of 0.11 (IQR −0.09 to 0.32) points of ALSFRS-R lost per month. Median serum NfL declined from 78.0 pg/ml (IQR 37.0–147.0 pg/ml; *n* = 23) to 36.0 pg/ml (IQR 22.0–65.0 pg/ml; *n* = 23; p = 0.02), median pNfH in CSF from 2226 pg/ml (IQR 1061–6138 pg/ml; *n* = 18) to 1151 pg/ml (IQR 521–2360 pg/ml; *n* = 18; p = 0.02). In the CSF, we detected a pleocytosis in 73% of patients (11 of 15) and an intrathecal immunoglobulin synthesis (IgG, IgM, or IgA) in 9 out of 10 patients. Two drug-related serious adverse events were reported.

**Interpretation:**

Consistent with the VALOR study and its Open Label Extension (OLE), our results confirm a reduction of NfL serum levels, and moreover show a reduction of pNfH in CSF. The therapy was safe, as no persistent symptoms were observed. Pleocytosis and Ig synthesis in CSF with clinical symptoms related to myeloradiculitis in two patients, indicate the potential of an autoimmune reaction.

**Funding:**

No funding was received towards this study.

**Research in context**

**Evidence before this study**

We searched PubMed for all publications on treatment with the antisense oligonucleotide (ASO) tofersen in patients with amyotrophic lateral sclerosis (ALS) carrying pathogenic variants of the *SOD1* gene published up to September 10th, 2023, using the terms “tofersen” in combination with each of the following terms: “amyotrophic lateral sclerosis” or “ALS”, “*SOD1* gene carriers”, “*SOD 1* mutation carriers”. After reviewing the abstracts, we identified 3 clinical trials and one case series reporting about the treatment with tofersen. Among the clinical trials there were two trials (phase II and III) investigating tofersen in patients with ALS and one in pre-symptomatic gene carriers. The case series investigated *n* = 6 patients over a mean treatment duration of 6.5 months.

**Added value of this study**

In this cohort study, we investigated and reported the effect of tofersen on clinical outcome parameters (ALSFRS-R, progression rate, quality of life, adverse events), laboratory findings in CSF, and biomarkers (NfL in serum, pNfH in CSF) in a multicenter cohort of ALS patients (*n* = 24) in a “real-world” setting. We confirmed a decrease of NfL serum levels under treatment with tofersen, but also demonstrated a reduction of pNfH in CSF. Median progression rate under tofersen treatment was 0.11 points of ALSFRS-R lost per month. We found that pleocytosis, elevated protein levels and intrathecal immunoglobulin synthesis were common findings in the CSF.

**Implications of all the available evidence**

Based on pre-existing evidence, treatment with tofersen in *SOD1*-ALS leads to a reduction in neurofilament levels (NfL). This study provides evidence that these findings can be extended to pNfH in CSF. Therefore, treatment with tofersen was shown to be an effective therapeutic approach. The observed alterations in the CSF suggest an autoimmune inflammation of the central nervous system, which necessitates special attention when treating patients with tofersen.

## Introduction

Amyotrophic lateral sclerosis (ALS) is a neurodegenerative disease characterized by progressive muscle weakness and a severely reduced life expectancy of about 2–5 years after onset by irreversible affection of the upper and lower motor neurons.^1^ A positive family history is found in about 5–10% of ALS patients.^2^^,^^3^ A pathogenic variant in the Cu/Zn superoxide dismutase (*SOD1*) gene was first described as a cause of familial ALS in 1993^4^ and can be detected in approximately 2% of sporadic^5^ and 11% of familial ALS patients in Central Europe.^6^ Pathogenic variants in the *SOD1* gene are the most common cause of the disease after *C9orf72* alterations in Europe and the most common mutations in Asia.^7^, ^8^, ^9^ More than 230 different mutations of *SOD1* with heterogenous clinical phenotypes have been described.^10^ Regarding pathogenesis, a toxic gain of function of mutant SOD1 protein has been proposed.^10^, ^11^, ^12^ However, a pathogenic loss of function, as can occur with homozygous mutations in children with motor neuron disease (MND), should also be discussed.^13^^,^^14^

For treatment of sporadic as well as genetic ALS, riluzole^15^ and (in some countries) edaravone^16^ and sodium phenylbutyrate/taurursodiol^17^ are the only approved drugs which nevertheless show limited effects. However, in ALS patients with (likely) pathogenic *SOD1* variants, intrathecal application of the antisense oligonucleotide (ASO) tofersen, which reduces the synthesis of SOD1 protein by RNase H-dependent degradation of *SOD1* messenger RNA, has recently shown very promising results.^18^^,^^19^ During the initial placebo-controlled phase I-III trial, a significant reduction of neurofilament light chain (NfL) in plasma compared to placebo suggested a disease-modifying effect,^19^ although clinical progression parameters such as ALS functional rating scale revised (ALSFRS-R) did not reach statistical significance after six months.^19^ However, in the Open Label Extension (OLE) phase of the VALOR study, Miller and colleagues reported after an overall observation period of 52 weeks a lower decrease in ALSFRS-R (difference 3.5 points) in participants, with early-start vs. those with delayed-start of treatment.^19^ Serious adverse events (SAEs), including autoimmune myelitis, meningitis, lumbar radiculopathy, intracranial hypertension, and papilledema, occurred in about 7% of patients recruited to the VALOR study.^19^

Based on these promising results, Early Access Programs (EAPs) were initiated in various countries, and later tofersen for the treatment of *SOD1*-ALS was approved by the U.S. Food and Drug Administration (FDA) in April 2023. In Germany, the first patients were included into the EAP in 03/2022, and concerted clinical data collection and biosampling were initiated within the German Motor Neuron Disease Network (MND-NET), a nationwide consortium of specialized ALS centers.

In this study, we analyzed the effect of tofersen on clinical parameters (ALSFRS-R, progression rate, muscle strength, and quality of life), biomarkers (phosphorylated neurofilament heavy chain (pNfH) in cerebrospinal fluid (CSF) as well as NfL in serum, creatine kinase (CK), and C-reactive protein (CRP) in serum), and safety (adverse events and CSF abnormalities) in a cohort of *n* = 24 ALS patients with (likely) pathogenic *SOD1* variants from ten centers participating in the German tofersen EAP.

## Methods

### Participants

Patients were enrolled from the MND-NET, a clinical and scientific network of 21 German motoneuron disease centres. We continuously collected data from 24 ALS patients with *SOD1* mutations from ten centers (University of Ulm, Charité Berlin, Bergmannsheil University Hospital Bochum, University of Bonn, University Hospital Carl Gustav Carus Dresden, University Hospital Erlangen, University Medicine Essen, Hannover Medical School, Helios Klinikum Krefeld, Ludwig Maximilians University Munich) who participated in the tofersen EAP. All patients were diagnosed with definite laboratory-supported, familial ALS according to the revised El Escorial criteria^20^ and were positively tested for a *SOD1* mutation, defined as causative for ALS based on classification of the variant as mutation grade 4 (likely pathogenic) or 5 (pathogenic) according to the American College of Medical Genetics and Genomics and the Association for Molecular Pathology (ACMG-AMP).^21^ The cohort contains data of three patients, which have been published previously by Meyer and colleagues.^22^

The inclusion criteria for participation in the EAP comprised the patients’ informed consent as well as the lack of contraindications to a lumbar puncture (oral anticoagulation etc.). Apart from riluzole, recruited patients did not receive any further disease-modifying drug therapy. Patients participating in the long-term extension of the placebo-controlled phase III tofersen study (VALOR) were excluded from the EAP and this study. The EAP included a dosing phase with intrathecal administration of 100 mg tofersen at day 1, 14 and 28, and a subsequent maintenance phase during which patients received up to 16 doses at intervals of about 28 (at least 21) days.

### Ethics

Informed consent was obtained from all participants. Biosampling of serum and CSF was conducted via participation in the MND-NET cohort study, for which separate informed consent was obtained. The study was approved by the local ethics committees of Ulm University (application number 19/12).

### Outcomes

Clinical data included the ALSFRS-R,^23^ progression rate (points of ALSFRS-R lost per month), survival, and muscle strength (arm abduction, arm flexion, hand extension, finger extension, finger spread, thumb abduction, hip flexion, knee extension, knee flexion, and foot extension) according to the Medical Research Council.^24^ Disease progression rate during the EAP was calculated by the formula (ALSFRS-R at first application—ALSFRS-R at last visit) divided by months between first application and last visit. The pre-baseline progression rate was calculated as (48—ALSFRS-R at first application) divided by months between onset and first application. Changes in disease and therapy related quality of life were assessed by the EuroQol Five Dimension Five Level Scale (EQ-5D-5L).^25^ During participation in the EAP, patients were asked about adverse events by the attending physician at each visit. Additionally, a safety telephone call was performed 24 h after each administration. All clinical outcome parameters were collected at all participating centers according to a uniform study protocol.

CSF parameters included leukocyte counts, total protein, lactate, oligoclonal IgG bands (OCB), immunoglobulin (Ig) synthesis, and pNfH. In blood, CRP,^26^, ^27^, ^28^ CK,^29^^,^^30^ and NfL were tested.^31^ For measurement of pNfH and NfL, CSF and blood samples from all participating centers were centrally analyzed at the CSF Laboratory of Ulm University.

### Analysis of CSF and blood samples

Mutation screening of the *SOD1* gene was performed using standard methods. Serum was obtained from peripheral blood by centrifugation (2000 g, 10 min) and stored within 2 h at −80 °C. Blood NfL concentrations were measured with the commercially available kits for the ELLA™ microfluidic system (Bio-Techne, Minneapolis, USA), the interassay coefficients of variation (CV) by analyzing two quality control samples (low and high levels of the analyte) on each cartridge were 9.3/6.1%, and the assay range was 2.7–10,290 pg/ml for NfL in serum. For CSF pNfH, the inter-assay CVs were 11.4/10.1%, and the assay range was 7.47–28,480 pg/ml.

CSF was obtained and analyzed as previously reported.^32^ Upon lumbar puncture, CSF samples were collected in polypropylene tubes and subject to analysis within 1 h from sampling. Leukocyte count was performed by a Fuchs–Rosenthal chamber. Total protein, albumin, IgG, IgA, and IgM were measured by standard immunochemical nephelometry in CSF and serum (Dade–Behring nephelometer analyzer (ProSpec), Marburg, Germany) using a polyclonal antibody for albumin, and IgG as well as a latex particle-amplified antibody reaction for IgA and IgM. The inter- and intra-assay variabilities were <10% for the method-dependent absolute levels as well as for the method-independent CSF/serum quotients of albumin, IgG, IgA, and IgM. CSF lactate was determined by a lactate–oxidase reaction (Greiner GmbH, Flacht, Germany).

OCB were determined by isoelectric focusing (IEF) on polyacrylamide gels followed by immunoblotting using an IgG-specific antibody staining.^33^ Briefly, CSF and serum samples from the same patient were adjusted for IgG concentrations and run on the same gel. Experienced technicians and neurologists with extensive experience in the field of CSF analysis independently evaluated and determined the subtype classification of the OCB^34^: Type 1- no oligoclonal IgG band, normal finding; Type 2- isolated OCB (2 or more IgG bands) in the CSF; Type 3- identical OCB (2 or more) in cerebrospinal fluid and serum, additionally (1 or more) isolated OCB in the CSF; Type 4- OCB with identical (mirror image) distribution in the CSF and serum; Type 5- monoclonal bands with identical distribution in the CSF and serum (usually suggesting a systemic gammopathy).

### Statistics

For descriptive statistics, median (IQR) are given. To analyze changes over time, the time of first tofersen application was defined as baseline, and the Wilcoxon signed rank test was applied. Two-sided 95% confidence intervals were calculated for median differences or differences of proportions, respectively. One-way ANOVA-analysis was performed for multiple comparisons. All statistical tests were performed at a two-sided level of alpha = 0.05. As this was an explorative study, except of Holm-Šídák's test for NfL and pNfH analyses, adjustment of p-values for multiple testing was not done, and all results were interpreted as hypothesis generating. Because of missing values, NfL and pNfH were analyzed by a mixed-effects model instead of ANOVA. Missing values were not replaced. Comparisons between groups with different progression rates were calculated with unpaired Student's t-test for continuous variables, Chi-Square-test for nominal variables and non-parametric Mann–Whitney U test for non-normally distributed variables.

Statistical analyses were performed using GraphPad Prism version 9.5.1 for Windows (GraphPad Software, San Diego, California USA).

### Role of funding source

This study was an investigator-initiated trial (IIT) within the framework of the German MND-NET. Biogen Inc. provided the drug and documents for the EAP, but was otherwise not involved in any aspect of this study, such as study design, data collection, data analyses, interpretation, or writing of the report.

## Results

### Demographic and clinical data at baseline

Overall, *n* = 24 patients with ALS carrying (likely) pathogenic variants in the *SOD1* gene and participating in the tofersen EAP were included in the study. In the median, participants received eight doses of tofersen (IQR 5–14) during a median observation period of 6.0 months (IQR 2.8–11.4). The first patient was included on 31st March 2022 and received 16 administrations. The end of the observation period for all patients, as reported in this manuscript, was the 30th April 2023. Fifty percent (*n* = 12) of participating patients were female and 50.0% (*n* = 12) were male. A positive family history of ALS was reported in 58.3% (*n* = 14) whereas 41.7% (*n* = 10) of cases were reported to be sporadic. Participating patients developed first symptoms 26.2 months (median, IQR 14.4–54.2 months) and were diagnosed with ALS 11.7 months (median, IQR 3.6–28.5 months) prior to EAP inclusion. At the time of the first administration of tofersen, the median age was 53.0 years (IQR 41.5–61.0 years). A spinal onset was reported in 95.8% (*n* = 23), and a bulbar onset in 4.2% (*n* = 1). Demographic and clinical data prior to the first application of tofersen are depicted in Table 1, and the spectrum of *SOD1* mutations is shown in Supplementary Table S1.

**Table 1:** Demographic and clinical data prior to the first application of tofersen.

Age (median, IQR)	53.0 (41.5–61.0) years (*n* = 24)
**Sex**	
Male	50.0% (*n* = 12)
Female	50.0% (*n* = 12)
**Onset**	
Spinal	95.8% (*n* = 23)
Bulbar	4.2% (*n* = 1)
**Type**	
Sporadic	41.7% (*n* = 10)
Familial	58.3% (*n* = 14)
**ALSFRS-R** (first visit) (median, IQR)	37.0 (29.8–41.8) (*n* = 24)
**Progression Rate** (onset to first visit) (ALSFRS-R points lost/month; median, IQR)	0.41 (0.20–0.83) (*n* = 24)
**Time onset to first administration** (median, IQR)	26.2 (14.5–54.2) months (*n* = 24)
**NfL in serum** (pg/ml) (median, IQR)	78 (37–147) (*n* = 23)
**pNfH in CSF** (pg/ml) (median, IQR)	2226 (1061–6138) (*n* = 18)

ALSFRS-R: ALS Functional Rating Scale-Revised, CSF: cerebrospinal fluid, NfL: neurofilament light chain, pNfH: phosphorylated neurofilament heavy chain.

### Clinical outcome parameters

**Fig. 1:**
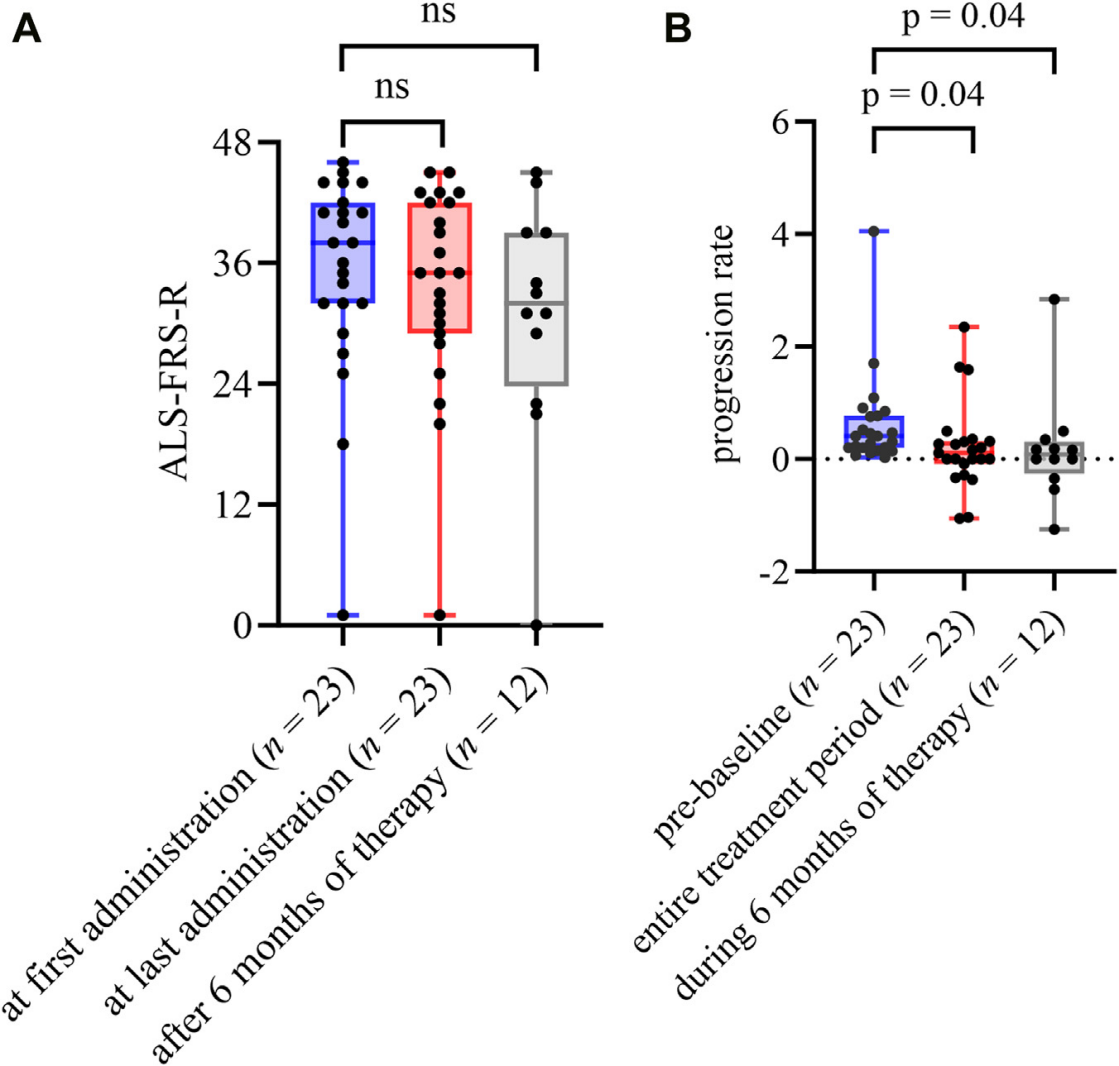
**ALSFRS-R total scores and progression rates.** Boxplots show median (IQR; minimum–maximum) of ALSFRS-R total scores and progression rates (ALSFRS-R points lost/month) prior to and during therapy with tofersen. **(A)** ALSFRS-R at first administration (blue), last administration (red), and after six months of therapy (grey) **(B)** progression rates pre-baseline (blue) compared to the entire treatment period (between first and last administration (red)) and during the first six months of therapy (grey) (first administration until six months of therapy). *Experimental units n = number, n = 23 comparisons first administration to last administration and pre-baseline to period between first and last administration, n = 12 comparisons to ALSFRS-R at six months of therapy and progression rate between first administration and six months of therapy. Median time from first to last ALSFRS-R 6.0 months (IQR 2.8–10.5 months). Changes over time were analyzed by Wilcoxon matched-pairs signed rank test. A p-value of ≤ 0.05 was regarded as statistically significant. ALSFRS-R: Amyotrophic lateral sclerosis functional rating scale revised. ns: not significant.*

**Fig. 2:**
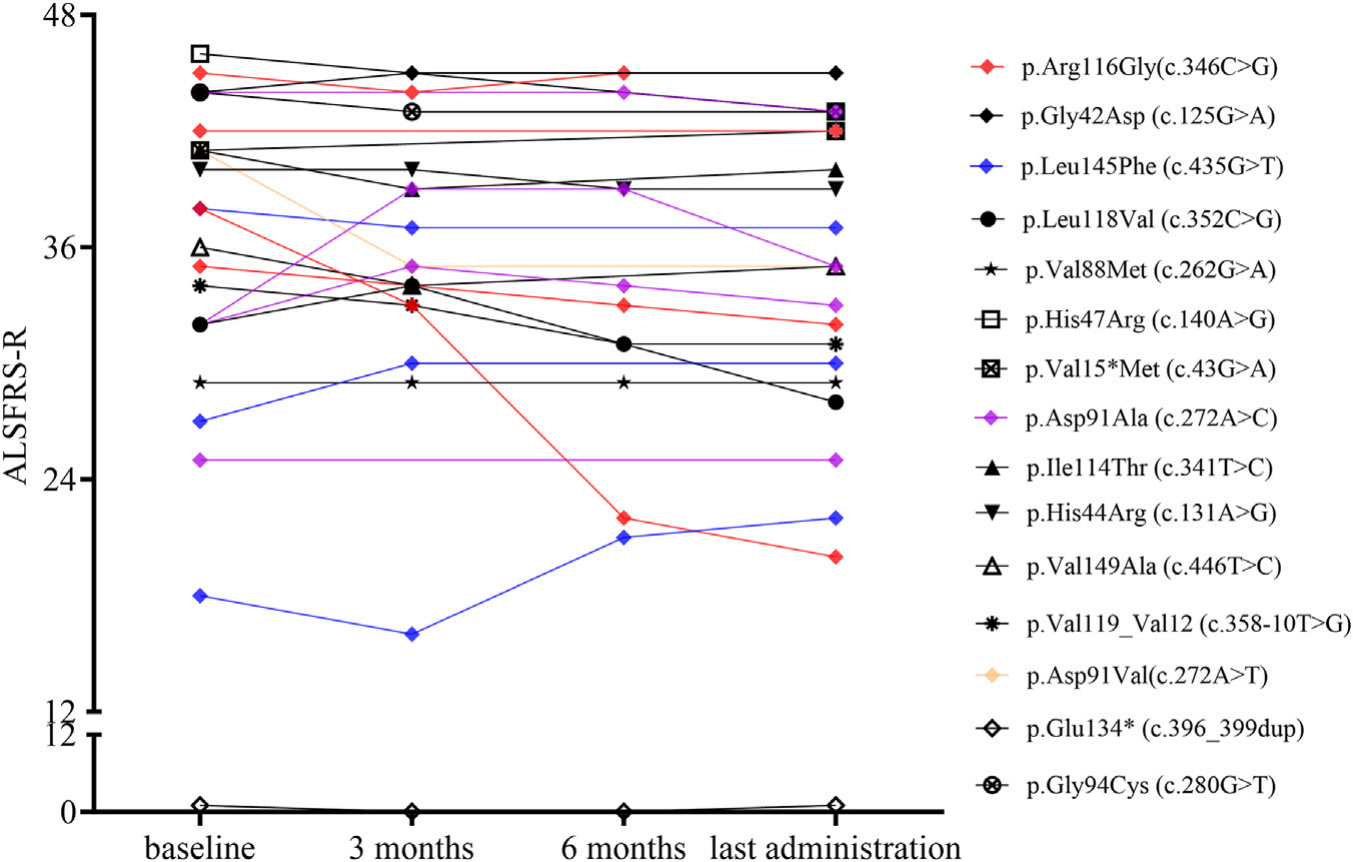
**ALSFRS-R on individual level.** Graphs show changes in ALSFRS-R on individual level prior to first administration of tofersen, after three and six months of therapy, and at the time of the last tofersen administration. *Experimental units n = number first and last administration n = 23, three months n = 18, six months n = 12. Time from first to last assessment of ALSFRS-R median 6.0 months (IQR 2.8–10.5 months). ALSFRS-R: Amyotrophic lateral sclerosis functional rating scale revised.*

None of the participating patients died during the observation period. One patient terminated tofersen therapy after the first administration due to personal decision, and data of this patient therefore could not be included in comparative follow-up analyses. Median ALSFRS-R at baseline (time of first tofersen application) was 38.0 (IQR 32.0–42.0; *n* = 23) and decreased to 35.0 (IQR 29.0–42.0; *n* = 23) at the last recorded tofersen administration, corresponding to a median progression rate of 0.11 (IQR −0.09 to 0.32; *n* = 23) points of ALSFRS-R lost per month. Therefore, during the whole observation period, but already during the time between first administration of tofersen and six months of treatment (median 0.08; IQR −0.26 to 0.31; *n* = 12), ALSFRS-R progression rate was significantly lower compared to the median progression rate prior to therapy (median 0.41; IQR 0.20–0.83; *n* = 12; p = 0.04 respectively) (Fig. 1). On an individual level, slower progression rates compared to pre-baseline were recorded in 17 patients, and faster progression rates in six patients during the study period (median ALSFRS-R loss per month 0.00; IQR −0.31 to 0.18; *n* = 17 vs. median 0.98; IQR 0.32–1.81; *n* = 6; p < 0.001) (Fig. 2). Patients who showed an increase in ALSFRS-R progression rate during tofersen therapy had a shorter disease duration prior to first administration, higher ALSFRS-R baseline values, and higher baseline NfL and pNfH levels compared to patients with a slowdown in the ALSFRS-R progression rate during tofersen treatment. Moreover, the observation period was shorter in these patients, implying that they have received fewer doses of tofersen. With the exception of pre-baseline ALSFRS-R progression rate, none of these differences was statistically significant, possibly due to the small sample size. Demographic and clinical data, biomarkers, and mutation spectrum in patients with decrease vs. increase of ALSFRS-R progression rate during tofersen therapy are depicted in Supplementary Table S2. In a subgroup of nine patients with available data, we did not observe any significant changes in muscle strength levels.

### Quality of life

Self-reported quality of life results measured by the EQ-5D-5L were available from *n* = 8 patients at the time of first administration of tofersen and at least at one further visit during the therapy. During the study period, patients showed almost stable results, generally indicating severe problems with regard to mobility and self-care but only slight to moderate levels of pain, discomfort, anxiety, and depression. The ability to perform work, study, housework, family, or leisure activities was reported to deteriorate from moderate to severe problems. When asked to indicate their current state of health on a visual analogue scale from 0 to 100 (0 indicating worst health and 100 best health), patients reported no significant changes during the observation period (median 55, IQR 41–69 at baseline vs. median 60, IQR 43–69 during therapy; p = 0.88). Only one patient, who had the highest pre-baseline progression rate (1.70 points of ALSFRS-R lost/month), showed a significant deterioration on the visual analogue scale (from 45 to 10).

### Serum/CSF biomarkers

**Fig. 3:**
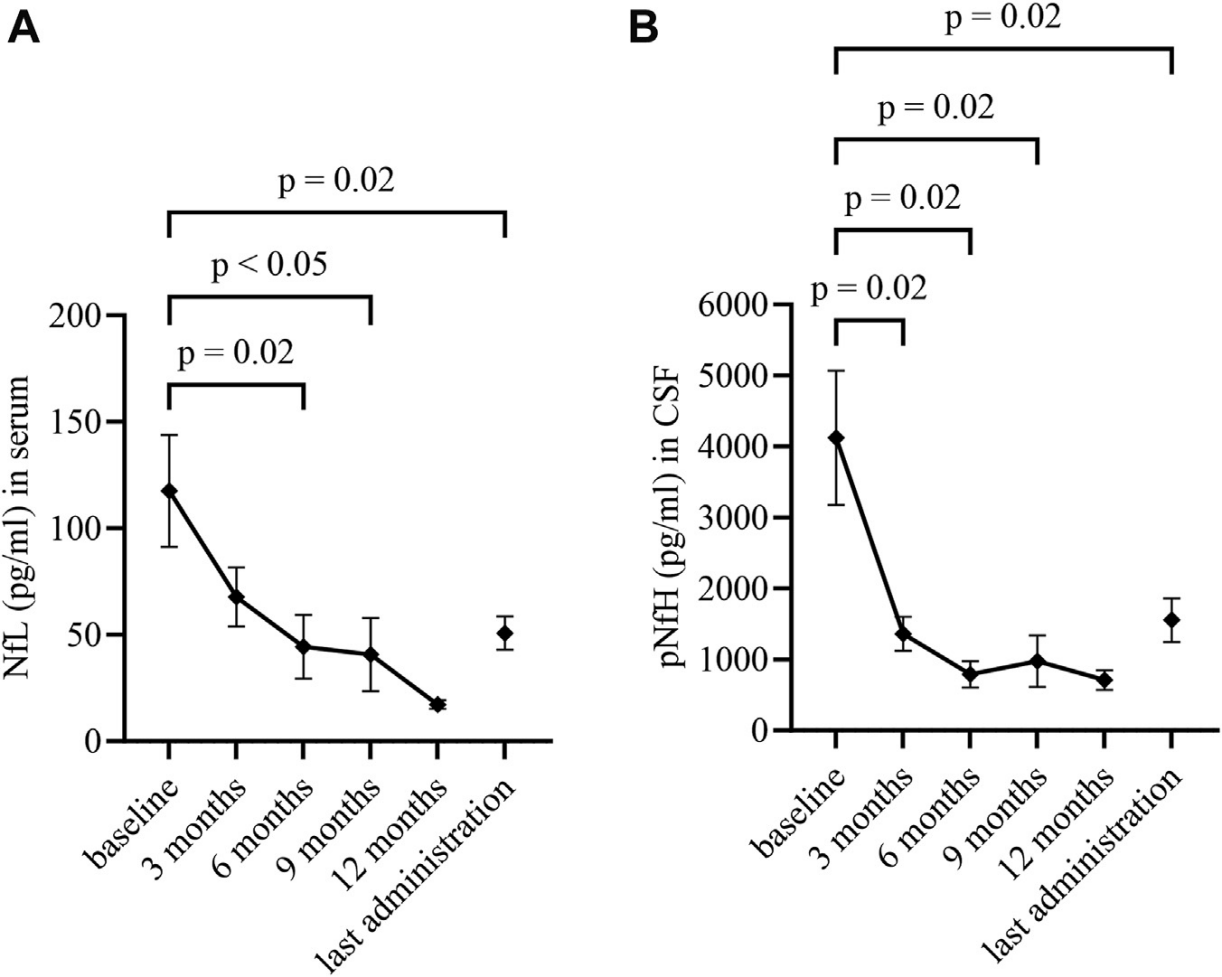
**Neurofilaments.** Graphs show mean (SEM) of NfL levels (pg/ml) in serum and p-NfH levels (pg/ml) in CSF prior to first administration of tofersen, after three, six, nine, twelve months of therapy and at the time of last the last tofersen administration. **(A)** NfL in serum (pg/ml) **(B)** p-NfH in CSF (pg/ml). *Experimental units n = number (A) first and last administration n = 23, three months n = 18, six months n = 11, nine months n = 8 and twelve months n = 4 (B) first and last administration n = 18, three months n = 15, six months n = 9, nine months n = 7 and twelve months n = 4. Time from first to last measurement of NfL in serum median 5.1 months (IQR 2.8–10.3 months) and of pNfH in CSF median 5.5 months (IQR 2.8–11.5 months). Changes over time were analyzed by one-way ANOVA-analysis and adjusting for multiple comparisons was performed by Holm-Šídák's test. A p-value of ≤ 0.05 was regarded as statistically significant. CSF: cerebrospinal fluid. NfL: neurofilament light chain. pNfH: phosphorylated neurofilament heavy chain.*

At baseline, median NfL levels in serum were 78.0 pg/ml (IQR 37.0–147.0 pg/ml; *n* = 23) and declined to 36.0 pg/ml (IQR 22.0–65.0 pg/ml; *n* = 23; p = 0.02) during the observation period (Fig. 3A). Significant reductions of NfL serum levels were already found after six months (23.0 pg/ml, IQR 17.0–38.0 pg/ml; *n* = 11, p = 0.02) and nine months (23.5 pg/ml, IQR 16.5–38.1; *n* = 8, p < 0.05) of therapy (Fig. 3A).

**Fig. 4:**
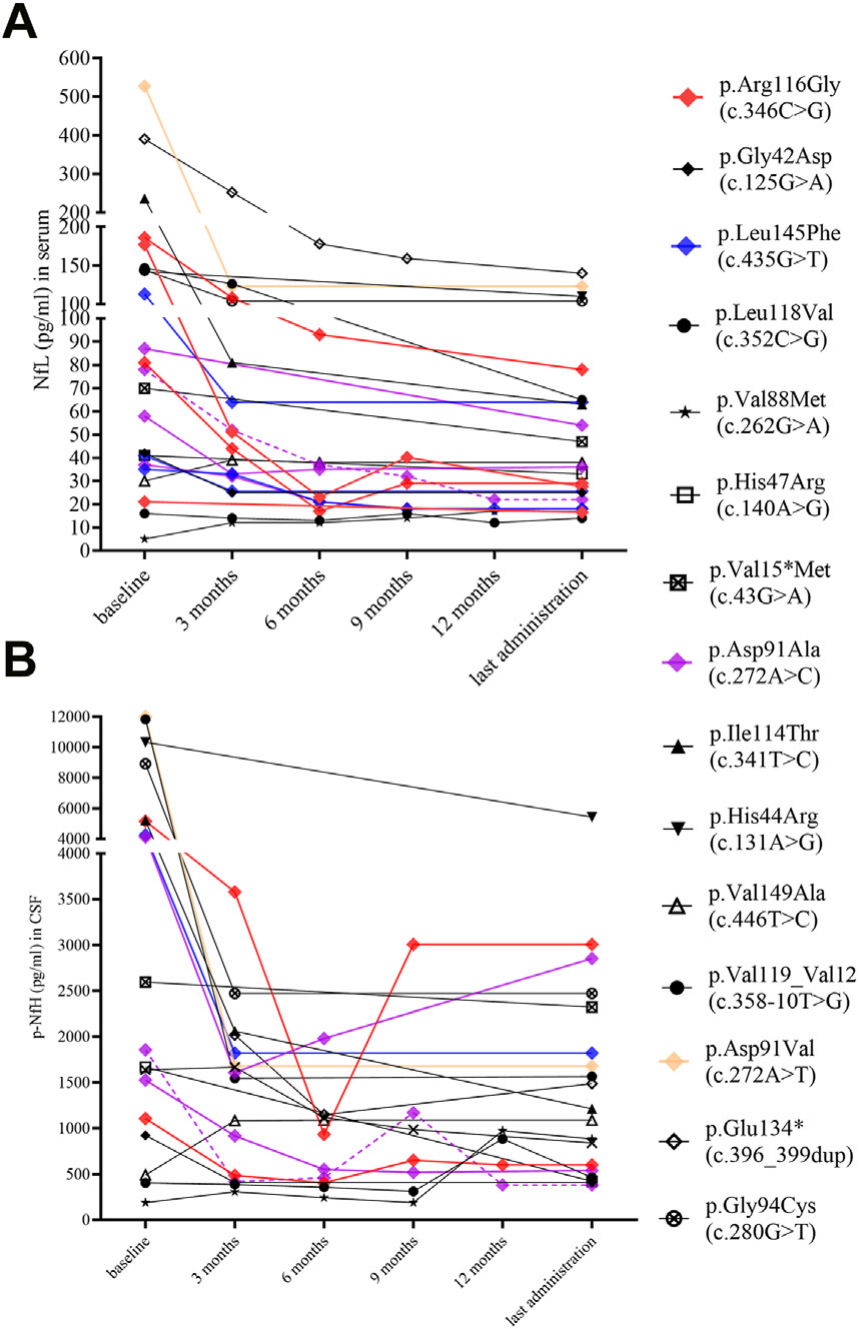
**Neurofilaments on individual level.** Graphs show changes in NfL levels (pg/ml) in serum and pNfH levels (pg/ml) in CSF on subject level prior to first administration of tofersen, after three, six, nine, twelve months of therapy and at the time of the last tofersen administration. **(A)** NfL in serum (pg/ml) **(B)** pNfH in CSF (pg/ml). *Experimental units n = number (A) first and last administration n = 23, three months n = 18, six months n = 11, nine months n = 8 and twelve months n = 4 (B) first and last administration n = 18, three months n = 15, six months n = 9, nine months n = 7 and twelve months n = 4. Time from first to last measurement of NfL in serum median 5.1 months (IQR 2.8–10.3 months) and of pNfH in CSF median 5.5 months (IQR 2.8–11.5 months). In p.Asp91Ala (c.272A > C), the dashed line indicates a heterozygous allele genotype. CSF: cerebrospinal fluid. NfL: neurofilament light chain. pNfH: phosphorylated neurofilament heavy chain.*

Likewise, median pNfH levels in CSF at baseline were 2226 pg/ml (IQR 1061–6138 pg/ml; *n* = 18) and declined to 1151 pg/ml (IQR 521–2360 pg/ml; *n* = 18) at last administration (p = 0.02) (Fig. 3B). Significant reductions of median pNfH CSF levels were already found after three (1545 pg/ml, IQR 417–1821 pg/ml; *n* = 15, p = 0.02), and as with NfL serum levels, after six (552 pg/ml, IQR 381–1104 pg/ml; *n* = 9, p = 0.02) and nine (651 pg/ml, IQR 312–1170 pg/ml; *n* = 7, p = 0.02) months of therapy (Fig. 3B). Longitudinal changes in NfL serum and pNfH CSF levels on a subject level are pictured in Fig. 4A and B.

Comparing the subgroups of patients with treatment periods longer and shorter than six months, it could be shown, that even in the subgroup of patients with a treatment period shorter than six months a significant decrease of NfL and pNfH was present (Supplementary Table S3).

Regarding CRP (*n* = 12) and CK (*n* = 10) serum levels, no significant changes were found during the observation period.

### Laboratory abnormalities

**Fig. 5:**
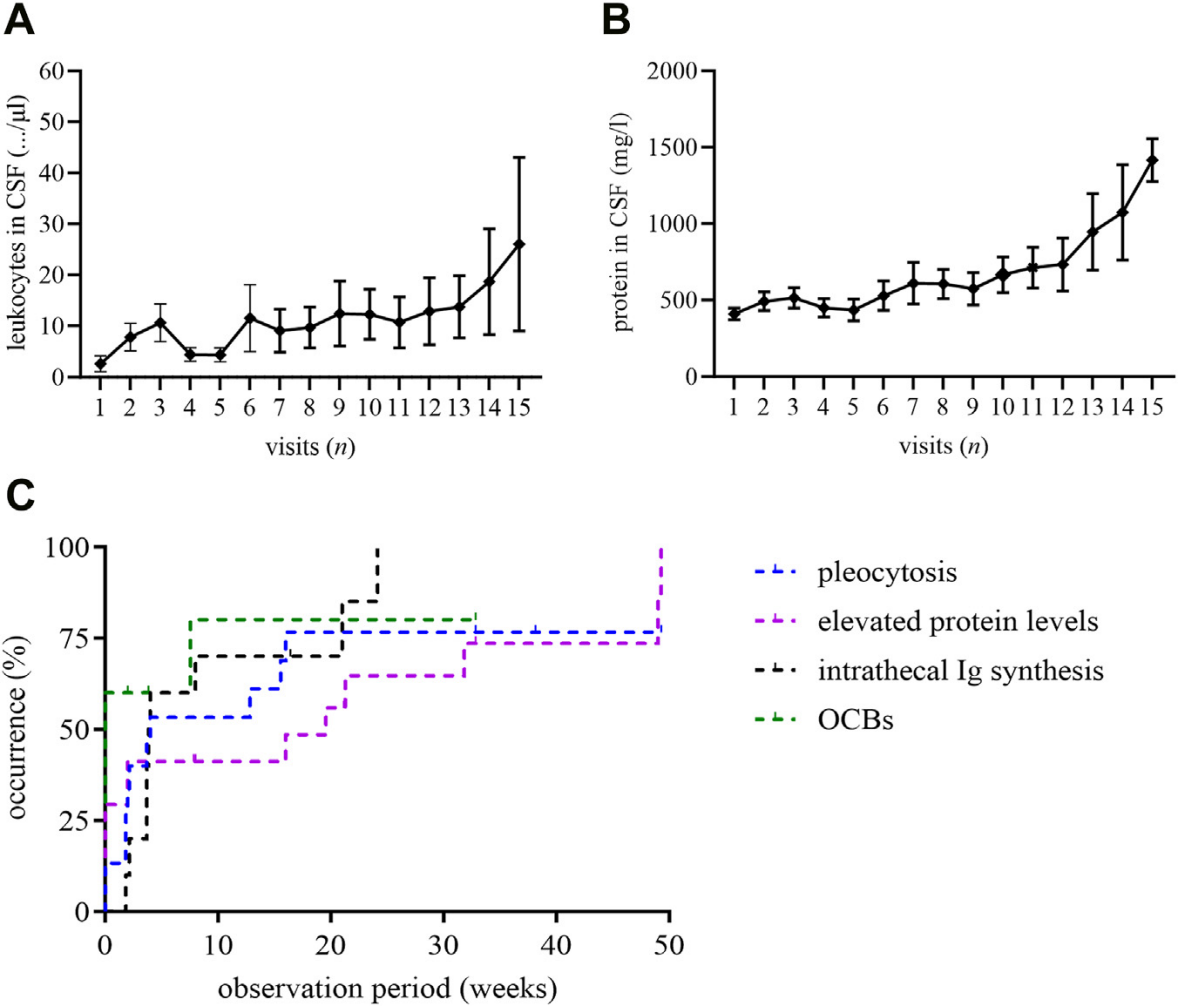
**CSF and serum findings per visit. (A)** mean (SEM) leukocyte counts in CSF **(B)** mean (SEM) protein levels in CSF **(C)** CSF and serum findings over time, graphs show proportion and time of first occurrence of pleocytosis ≥5 leukocytes/μl in CSF (blue), elevated protein levels >500 mg/l in CSF (violet), intrathecal immunoglobulin synthesis (black) and oligoclonal bands (green). *Experimental units n = number (A) n = 18 (B) n = 19 (C) (blue) n = 15 (violet) n = 17 (black) n = 10 (green) n = 10. First occurrence of pleocytosis median 3.7 weeks (IQR 1.9–14.2 weeks, n = 9; additionally pre-existing n = 2), first occurrence of elevated protein levels median 20.4 weeks (IQR 5.5–44.7 weeks, n = 8; additionally pre-existing n = 5), first occurrence of intrathecal immunoglobulin synthesis median 3.9 weeks (IQR 2.9–14.5 weeks, n = 9), first occurrence of oligoclonal bands (OCBs) in one patient after 7.6 weeks (additionally pre-existing n = 6). CSF: cerebrospinal fluid. Ig: immunoglobulin. OCB: oligoclonal bands.*

In CSF, a pleocytosis of ≥5 leukocytes/μl was found in 73% (11 of 15) patients with available data during the therapy, ranging from 8 to 56 leukocytes/μl. This increase of cells was detected after a median of 3 (IQR 2–6) administrations (median 3.7 weeks, IQR 1.9–14.2; Fig. 5A–C; Supplementary Table S4). One patient with a pronounced pleocytosis of 56/μl at the 6th administration showed clinical signs of autoimmune myeloradiculitis. Pleocytosis was accompanied by an increase in total protein in 10 cases. An increase in CSF total protein occurred after a median of 7 (IQR 3–13) administrations (median 20.4 weeks, IQR 5.5–44.7; Fig. 5B and C; Supplementary Table S4). Overall, the median maximum CSF leukocyte count was 15.0/μl (IQR 4.7–45.0; *n* = 15; Supplementary Table S4). No pathological changes in lactate levels were recorded. During the observation period, 90% (9/10) of patients with available data developed an intrathecal IgM, IgG, and/or IgA synthesis. Intrathecal IgM synthesis was found after a median of 3 visits (IQR 3–6; median 3.9 weeks, IQR 2.9–14.5), while intrathecal IgG synthesis was present in 2 patients after 3 and 9 administrations, respectively. In one of these two cases, intrathecal immunoglobulin synthesis was accompanied by appearance of new oligoclonal bands, whereas in the other case, oligoclonal bands were already pre-existing (Fig. 5C; Supplementary Table S4).

### Adverse events

Two potentially drug-related serious adverse events were reported during the observation period. One patient was diagnosed with autoimmune myeloradiculitis after the sixth dose of tofersen and discontinued the EAP. Another patient experienced transient lower limb weakness after each of the three doses (one month of therapy) and discontinued treatment. In both patients the symptoms were completely reversible, but in the first patient only after immunomodulatory treatment. Both patients decided on their own not to continue treatment after these side effects. In the second patient, there was no objective medical reason for discontinuation. Another patient discontinued tofersen therapy due to personal reasons after one administration of tofersen without any prior complications or adverse events. Lumbar back pain, headache, transient radicular complaints of the lower extremities, and dizziness were classified as procedure-related. During the observation period, no other drug-related adverse events were reported.

## Discussion

In this study, we investigated the effects of tofersen treatment in patients with *SOD1*-ALS for the first time in a “real-world” setting, i.e., including and analyzing all *SOD1* patients independent from the VALOR inclusion/exclusion criteria. As we found a significant reduction of NfL in serum, we were able to corroborate the results of the placebo-controlled phase III trial (VALOR study and its OLE) in this heterogeneous cohort. Moreover, we found beneficial signals in additional outcome parameters such as pNfH. Of note, CSF analysis suggested a clinically silent autoimmune inflammation of the central nervous system as a common finding under treatment with tofersen. In accordance with the pivotal VALOR trial,^19^ we found a reduction of the established ALS biomarker NfL in serum during tofersen treatment, suggesting that tofersen is a disease-modifying treatment. Moreover, we showed for the first time that tofersen treatment also reduced pNfH in CSF, further strengthening the evidence for the efficacy of tofersen. During the observation period, patients showed a median progression rate of 0.11 points of ALSFRS-R lost per month. As a small subgroup of six patients showed an increase in ALSFRS-R progression rate during tofersen therapy, it has to be discussed whether this group actually represents “non-responders” or whether the treatment duration was still too short in these patients to demonstrate a clinical effect. We found that these six patients had higher baseline NfL and pNfH levels as well as a shorter disease duration compared to responders, suggesting a more aggressive disease course. As the median duration of therapy in these patients was shorter compared to the responders, a reassessment after a longer observation period would be interesting.

Furthermore, patients reported a stable health-related quality of life during EAP participation, which is consistent with the VALOR study and its OLE.^19^ Depressive symptoms and pain have been reported to be related to poorer quality of life in ALS.^35^ Anxiety/depression and pain/discomfort have been described to be the most stable domains in EQ-5D-5L in the pre-existing literature,^36^ while quality of life seems not to correlate with physical impairment.^37^ As anxiety/depression and pain/discomfort only showed minor changes under tofersen treatment, this might partly explain the stable results in EQ-5D-5L total score. Moreover, it can be hypothesized, that the anticipation of a positive treatment effect of tofersen may also contribute to a stable mood and therefore better quality of life. Overall, it cannot be conclusively clarified to what extent tofersen contributed to the unchanged quality of life.

The rate of drug-related SAEs, most notably one patient (4.2%) suffering from autoimmune myeloradiculitis, was lower compared to previously reported cases.^19^ In another case of transient weakness of the lower extremities it remains unclear whether these symptoms were a side effect of tofersen therapy. In both patients the symptoms were completely reversible, but in the first patient only after immunomodulatory treatment. Thus, overall, we can conclude that tofersen was generally well tolerated.

Of note, we observed that clinically asymptomatic pleocytosis and protein elevation in CSF, indicating an autoimmune inflammation of the central nervous system, were present in the majority of patients. In several cases, these changes were accompanied by intrathecal immunoglobulin synthesis, including all three classes with predominant IgM and oligoclonal IgG bands. Immunological reactions in CSF have previously also been reported in several cases of patients with spinal muscular atrophy (SMA) under treatment with the ASO nusinersen.^38^^,^^39^ Therefore, we think that the immunological mechanisms underlying ASO treatment require further attention and investigation. Although the observed phenomena seem to be rarely accompanied by relevant clinical symptoms, we believe that careful monitoring of CSF findings and clinical symptoms is necessary.

Moreover, since pathogenic loss of function has been discussed as a possible cause of MND in children with homozygous mutations,^13^^,^^14^ it would be interesting to clarify whether positive effects of tofersen can be reproduced in this subgroup, or whether tofersen leads to worsening of symptoms by reducing SOD1 protein levels in these patients. Our study is not without limitations. Since tofersen is a new drug, which has not yet been approved in Europe, and since it has only been available for open treatment in Germany since March 2022 in the EAP, the number of treated cases and the observation period are limited.

Included patients were heterogeneous with regard to mutations in the *SOD1* gene, disease duration, observation time, and number of follow-up visits, making it more difficult to compare the data. Furthermore, as opposed to the pivotal study,^19^ evaluated parameters could not be compared to placebo. Therefore, a placebo effect has to be considered. Regarding the evaluation of outcome parameters, pre-baseline progression rates relied on anamnestic information, which is prone to bias. As the median observation period during tofersen treatment (6.0 months) was shorter compared to the pre-baseline period (26.2 months), the comparison of pre-baseline and on-treatment ALSFRS-R slopes may favor the on-treatment slope by default, as the observation period might have been too short to detect smaller clinical changes. Therefore, in our study, it was not possible to evaluate alterations in disease progression rate during tofersen therapy compared to pre-baseline. Overall three patients were lost to ALSFRS-R follow-up due to adverse events (autoimmune myeloradiculitis, subjective transient weakness of lower limbs) or due to personal reasons. Available data on muscle strength levels were limited to a small subgroup of nine patients. Therefore, an impact of tofersen on muscle strength could not be adequately assessed in this study and needs to be re-examined in a larger cohort.

Despite these limitations, we were still able to obtain clinically relevant findings, corroborating the positive findings obtained in the initial phase III study and its OLE^19^ in a real-life clinical routine setting over a significant observation period and expanding the pre-existing results by obtaining positive signals on pNfH in CSF during the therapy. The German tofersen EAP had no limitations regarding the inclusion of patients with specific mutations and/or progression rates. As mutations associated with comparatively benign disease courses are prevalent in Central Europe, slow progressors might be overrepresented compared to other geographic regions, as signified by a low median pre-baseline progression rate.

In summary, the findings of this study suggest that treatment of *SOD1*-ALS patients with intrathecal administration of tofersen is an effective therapeutic approach. In general, we found the therapy to be safe, although one single case of myeloradiculitis with a need for immunomodulatory treatment did occur. Further evaluation of frequently observed alterations in CSF, such as pleocytosis, elevated protein levels and, in some cases, CSF-specific oligoclonal IgG bands and intrathecal immunoglobulin synthesis, suggesting an autoimmune inflammation of the central nervous system, will be required in the future.

## Contributors

MW, JD: conceptualisation, data curation, formal analysis, investigation, methodology, validation, visualisation, writing—original draft, and writing—review & editing. ACL: conceptualisation, investigation, methodology, project administration, resources, supervision, writing—review & editing. DB: conceptualisation, formal analysis, investigation, methodology, project visualisation, and writing—review & editing. HT: conceptualisation, investigation, methodology, supervision, and writing—review & editing. KG, EF, AK: data curation, and writing—review & editing. ZE, ÖP, ZU, KK, KM, UWei, CH, JS, AF, KM, RS, FB, TS, MR, AG, SP, JG, TK, PR, FS, THag, UWey, RG, MV, MJ, THaa, PW, IV, JH, JC, JHW, PS, PK, TM, WPR, SW, MS: investigation, and writing—review & editing. MW and JD directly accessed and verified the underlying data reported in the manuscript. All authors read and approved the final version of the manuscript.

## Data sharing statement

Individual participant data that underlie the results reported in this article, after de-identification (text, tables, and figures) as well as the study protocol will be available. Data will be available beginning 3 months and ending 5 years following article publication. Data will be shared with researchers who provide a methodologically sound proposal. Data will be shared for analyses to achieve the aims in the approved proposal. Proposals should be directed to albert-c.ludolph@uni-ulm.de; to gain access, data requestors will need to sign a data access agreement.

## Declaration of interests

MW reports no competing interests. JD reports speaker honoraria from Biogen Inc. ZE, ÖP, KK, KM, UWei, CH, JS, KM, RS*,* FB, TS, KG, EF, AK, TK, PR, UWey, MV, MJ, THaa, IV, JH, JC, JHW, PS, PK, WPR and SW report no competing interests. DB reports stocks from Ionis Pharmaceuticals (100 stocks). ZU reports financial support from Biogen, Roche and Novartis for SMArtCARE data collection (site Ulm). He received grants, consulting fees for advisory boards, lectures, presentations, manuscript writing, educational events, and support for travel expenses from Biogen. He received payment for expert testimony (Case report, no guideline development) from Thieme and Biogen. AF reports grants or contracts from DFG (German Research Foundation, Project funding not related to this manuscript). MR received lecture honoraria from Zambon. AG reports grants from German Society of Cryobanks (GDK e.V.). SP reports grants or contracts from the German Israeli Foundation, the German Neuromuscular Society, and the Neurodegenerative Research Inc. SP reports consulting fees from Amylyx, Biogen, ITF Pharma, Roche, Zambon and Ferrer, and payment or honoraria for lectures, presentations, speakers bureaus, manuscript writing or educational events from Amylyx, Biogen, ITF Pharma, Roche, and Zambon. SP received support for travel and/or attend meetings from Amylyx, Zambon, and PTC Therapeutics. JG reports a startup grant from the University foundation of the University of Lübeck. He received consultation fees from Amylyx, Clene Nanomedicine for consultancy on drug development in ALS and payment for expert testimony on EU and national level from Amylyx. JG participated on Data Safety Monitoring Boards or Advisory Boards for UCB, ITF Pharma, and Ferrer. FS reports participation on Advisory Boards for Amylyx, Alnylam, and Alexion. THag reports grants and honoraria from Biogen, Roche, Novartis (all for SMA-Research), Sanofi Genzyme (Neuromuscular Diseases Research), and payment or honoraria for lectures, presentations, speakers bureaus, manuscript writing or educational events from Alexion, Argenx (both for Myasthenia gravis), Biogen, Novartis, Sanofi Genzyme, Roche (SMA) and Amylyx (ALS). THag participated on Data Safety Monitoring Boards or Advisory Boards for Biogen, Novartis, Roche (all for SMA), Alexion, Argenx, UCB (all for Myasthenia gravis), Amylyx (for ALS), Sanofi Genzyme (for LOPD). RG reports honoraria for lectures from Biogen, Roche, and Zambon. He participated on Advisory Boards for Biogen, Roche, Zambon, and ITF Pharma. RG reports research support from Biogen and Zambon. PW served on Advisory Boards for Zambon, and ITF Pharma. PK reports consulting fees from Biogen. TM is on the Advisory Board of Biogen and received consulting fees from Biogen. MS reports consulting fees from Alexion, Bayer, Biogen, Bristol-Myers-Squibb/Celgene, Merck, Horizon, Roche, and Sanofi Genzyme. MS reports payment or honoraria for lectures, presentations, speakers bureaus, manuscript writing or educational events from Alexion, Horizon, Roche, and Sanofi Genzyme and support for attending meetings and/or travel from Alexion, Celgene, Horizon, Roche, and Sanofi Genzyme. MS participated on Data Safety Monitoring Board or Advisory Board for Alexion, Bayer, Biogen, Bristol-Myers-Squibb, Merck, Horizon, Roche, and Sanofi Genzyme. HT reports grants or contracts from Sanofi Genzyme, German Multiple Sclerosis Society (DMSG), AMSEL Ursula-Späth-Stiftung, Bayern-DMSG, Deutsche MS-Stiftung, Ministry of Science, Research and Arts of the State Baden-Württemberg (MWK-BW), Chemische Fabrik Karl Bucher. HT received consulting fees from Merck, Novartis, Roche and received payment or honoraria for lectures, presentations, speakers bureaus, manuscript writing or educational events from Alexion, Bayer, Biogen, Celgene, GSK, Janssen, Merck, Novartis, Roche, Sanofi Genzyme, Siemens, TEVA, Viatris. HT reports support for attending meetings and/or travel from Janssen, Merck, Novartis, Roche, Sanofi Genzyme. He reports leadership or fiduciary role in DGLN, DMSG and AMSEL. ACL is a member of Advisory Boards of Roche Pharma AG, Biogen, Alector and Amylyx. He received compensation for talks from Biologix, the German Society of Neurology, Biogen, Springer Medicine, Amylyx and the company Streamed Up! GmbH. He is involved in trials which are sponsored by Amylyx, Ferrer International, Novartis Research and Development, Mitsubishi Tanabe, Apellis Pharmaceuticals, Alexion, Orion Pharma, the European Union, BMBF, Biogen and Orphazyme, Ionis Pharmaceuticals, QurAlis and Alector.

## Appendix A Supplementary data

Supplementary Table S4

Worksheet Tofersen

## References

[1] Masrori P., Van Damme P. (2020). Amyotrophic lateral sclerosis: a clinical review. Eur J Neurol.

[2] Chiò A., Calvo A., Moglia C., Mazzini L., Mora G., PARALS study group (2011). Phenotypic heterogeneity of amyotrophic lateral sclerosis: a population based study. J Neurol Neurosurg Psychiatry.

[3] Rosenbohm A., Peter R.S., Erhardt S. (2017). Epidemiology of amyotrophic lateral sclerosis in Southern Germany. J Neurol.

[4] Rosen D.R., Siddique T., Patterson D. (1993). Mutations in Cu/Zn superoxide dismutase gene are associated with familial amyotrophic lateral sclerosis. Nature.

[5] Ruf W.P., Boros M., Freischmidt A. (2023). Spectrum and frequency of genetic variants in sporadic amyotrophic lateral sclerosis. Brain Commun.

[6] Müller K., Brenner D., Weydt P. (2018). Comprehensive analysis of the mutation spectrum in 301 German ALS families. J Neurol Neurosurg Psychiatry.

[7] DeJesus-Hernandez M., Mackenzie I.R., Boeve B.F. (2011). Expanded GGGGCC hexanucleotide repeat in noncoding region of C9ORF72 causes chromosome 9p-linked FTD and ALS. Neuron.

[8] Renton A.E., Majounie E., Waite A. (2011). A hexanucleotide repeat expansion in C9ORF72 is the cause of chromosome 9p21-linked ALS-FTD. Neuron.

[9] Zou Z.Y., Zhou Z.R., Che C.H., Liu C.Y., He R.L., Huang H.P. (2017). Genetic epidemiology of amyotrophic lateral sclerosis: a systematic review and meta-analysis. J Neurol Neurosurg Psychiatry.

[10] Huai J., Zhang Z. (2019). Structural properties and interaction partners of familial ALS-associated SOD1 mutants. Front Neurol.

[11] Tafuri F., Ronchi D., Magri F., Comi G.P., Corti S. (2015). SOD1 misplacing and mitochondrial dysfunction in amyotrophic lateral sclerosis pathogenesis. Front Cell Neurosci.

[12] Bunton-Stasyshyn R.K., Saccon R.A., Fratta P., Fisher E.M. (2015). SOD1 function and its implications for amyotrophic lateral sclerosis Pathology: new and renascent themes. Neuroscientist.

[13] Andersen P.M., Nordström U., Tsiakas K. (2019). Phenotype in an infant with *SOD1* homozygous truncating mutation. N Engl J Med.

[14] Brenner D., Freischmidt A. (2022). Update on genetics of amyotrophic lateral sclerosis. Curr Opin Neurol.

[15] Petrov D., Mansfield C., Moussy A., Hermine O. (2017). ALS clinical trials review: 20 years of failure. Are we any closer to registering a new treatment?. Front Aging Neurosci.

[16] Writing Group; Edaravone (MCI-186) ALS 19 Study Group (2017). Safety and efficacy of edaravone in well defined patients with amyotrophic lateral sclerosis: a randomised, double-blind, placebo-controlled trial. Lancet Neurol.

[17] Paganoni S., Macklin E.A., Hendrix S. (2020). Trial of sodium phenylbutyrate-taurursodiol for amyotrophic lateral sclerosis. N Engl J Med.

[18] Miller T., Cudkowicz M., Shaw P.J. (2020). Phase 1-2 trial of antisense oligonucleotide tofersen for *SOD1* ALS. N Engl J Med.

[19] Miller T.M., Cudkowicz M.E., Genge A. (2022). Trial of antisense oligonucleotide tofersen for *SOD1* ALS. N Engl J Med.

[20] Brooks B.R., Miller R.G., Swash M., Munsat T.L. (2000). World federation of Neurology research group on motor neuron diseases. El escorial revisited: revised criteria for the diagnosis of amyotrophic lateral sclerosis. Amyotroph Lateral Scler Other Motor Neuron Disord.

[21] Richards S., Aziz N., Bale S. (2015). Standards and guidelines for the interpretation of sequence variants: a joint consensus recommendation of the American college of medical genetics and genomics and the association for molecular pathology. Genet Med.

[22] Meyer T., Schumann P., Weydt P. (2023). Neurofilament light-chain response during therapy with antisense oligonucleotide tofersen in SOD1-related ALS: treatment experience in clinical practice. Muscle Nerve.

[23] Cedarbaum J.M., Stambler N., Malta E. (1999). The ALSFRS-R: a revised ALS functional rating scale that incorporates assessments of respiratory function. BDNF ALS Study Group (Phase III). J Neurol Sci.

[24] Compston A. (2010). Aids to the investigation of peripheral nerve injuries. Medical research council: nerve injuries research committee. His Majesty's stationery office: 1942; pp. 48 (iii) and 74 figures and 7 diagrams; with aids to the examination of the peripheral nervous system. By Michael O'Brien for the guarantors of brain. Saunders Elsevier: 2010; pp. [8] 64 and 94 figures. Brain.

[25] Herdman M., Gudex C., Lloyd A. (2011). Development and preliminary testing of the new five-level version of EQ-5D (EQ-5D-5L). Qual Life Res.

[26] Kharel S., Ojha R., Preethish-Kumar V., Bhagat R. (2022). C-reactive protein levels in patients with amyotrophic lateral sclerosis: a systematic review. Brain Behav.

[27] Lunetta C., Lizio A., Maestri E. (2017). Serum C-reactive protein as a prognostic biomarker in amyotrophic lateral sclerosis. JAMA Neurol.

[28] Batty G.D., Kivimäki M., Frank P., Gale C.R., Wright L. (2023). Systemic inflammation and subsequent risk of amyotrophic lateral sclerosis: prospective cohort study. medRxiv.

[29] Ito D., Hashizume A., Hijikata Y. (2019). Elevated serum creatine kinase in the early stage of sporadic amyotrophic lateral sclerosis. J Neurol.

[30] Rafiq M.K., Lee E., Bradburn M., McDermott C.J., Shaw P.J. (2016). Creatine kinase enzyme level correlates positively with serum creatinine and lean body mass, and is a prognostic factor for survival in amyotrophic lateral sclerosis. Eur J Neurol.

[31] Steinacker P., Feneberg E., Weishaupt J. (2016). Neurofilaments in the diagnosis of motoneuron diseases: a prospective study on 455 patients. J Neurol Neurosurg Psychiatry.

[32] Jesse S., Brettschneider J., Süssmuth S.D. (2011). Summary of cerebrospinal fluid routine parameters in neurodegenerative diseases. J Neurol.

[33] Halbgebauer S., Haußmann U., Klafki H., Tumani H., Wiltfang J., Otto M. (2015). Capillary isoelectric focusing immunoassay as a new nanoscale approach for the detection of oligoclonal bands. Electrophoresis.

[34] Andersson M., Alvarez-Cermeño J., Bernardi G. (1994). Cerebrospinal fluid in the diagnosis of multiple sclerosis: a consensus report. J Neurol Neurosurg Psychiatry.

[35] Pizzimenti A., Aragona M., Onesti E., Inghilleri M. (2013). Depression, pain and quality of life in patients with amyotrophic lateral sclerosis: a cross-sectional study. Funct Neurol.

[36] Peseschkian T., Cordts I., Günther R. (2021). A nation-wide, multi-center study on the quality of life of ALS patients in Germany. Brain Sci.

[37] Lulé D., Häcker S., Ludolph A., Birbaumer N., Kübler A. (2008). Depression and quality of life in patients with amyotrophic lateral sclerosis. Dtsch Arztebl Int.

[38] Wurster C.D., Koch J.C., Cordts I. (2019). Routine cerebrospinal fluid (CSF) parameters in patients with spinal muscular atrophy (SMA) treated with nusinersen. Front Neurol.

[39] Müschen L.H., Osmanovic A., Binz C. (2021). Cerebrospinal fluid parameters in antisense oligonucleotide-treated adult 5q-spinal muscular atrophy patients. Brain Sci.

