# Supplementary material for: Effects of tofersen treatment in patients with SOD1-ALS in a “real-world” setting – a 12-month multicenter cohort study from the German early access program
Source: eClinicalMedicine. 2024 Feb 15;69:102495. doi: 10.1016/j.eclinm.2024.102495 (PMC10878861; doi:10.1016/j.eclinm.2024.102495)
Supplement: Supplementary Table S1 [file mmc1.docx]

**Supplementary table 1: *SOD1* mutation spectrum**

| **Mutation** | **Allele genotype** | **Heredity** | **ACMG-AMP class** | **(*n*)** |
| --- | --- | --- | --- | --- |
| **p.Val15*Met**_1_ **(c.43G>A**_2_**)** | heterozygous | sporadic | likely pathogenic | 1 |
| **p.Gly42Asp**_1_ **(c.125G>A**_2_**)** | heterozygous | familial | likely pathogenic | 1 |
| **p.His44Arg**_1_ **(c.131A>G**_2_**)** | heterozygous | familial | likely pathogenic | 1 |
| **p.His47Arg**_1_ **(c.140A>G**_2_**)** | heterozygous | familial | pathogenic | 1 |
| **p.Val88Met**_1_ **(c.262G>A**_2_**)** | heterozygous | sporadic | pathogenic | 1 |
| **p.Asp91Ala**_1_ **(c.272A>C**_2_**)** | homozygous (*n*=3) | familial (*n*=1) | pathogenic | 4 |
|  | heterozygous (*n*=1) | sporadic (*n*=3) |  |  |
| **p.Asp91Val**_1_ **(c.272A>T**_2_**)** | homozygous (*n*=1) | familial (*n*=2) | pathogenic | 2 |
|  | heterozygous (*n*=1) |  |  |  |
| **p.Gly94Cys**_1_ **(c.280G>T**_2_**)** | heterozygous | familial | pathogenic | 1 |
| **p.Ile114Thr**_1_ **(c.341T>C**_2_**)** | heterozygous | sporadic | likely pathogenic | 1 |
| **p.Arg116Gly**_1_ **(c.346C>G**_2_**)** | heterozygous (*n*=4) | familial (*n*=4) | likely pathogenic | 4 |
| **p.Leu118Val**_1_ **(c.352C>G**_2_**)** | heterozygous | sporadic | likely pathogenic | 1 |
| **p.Val119_Val12**_1_ **(c.358-10T>G**_2_**)** | heterozygous | sporadic | likely pathogenic | 1 |
| **p.Glu134***_1_ **(c.396_399dup**_2_**)** | heterozygous | familial | likely pathogenic | 1 |
| **p.Leu145Phe**_1_ **(c.435G>T**_2_**)** | heterozygous (*n*=3) | familial (*n*=1) | pathogenic | 3 |
|  |  | sporadic (*n*=2) |  |  |
| **p.Val149Ala**_1_ **(c.446T>C**_2_**)** | heterozygous | familial | likely pathogenic | 1 |

*_1_NP_000445.1, _2_NM_000454.5*
