# Supplementary material for: Effects of tofersen treatment in patients with SOD1-ALS in a “real-world” setting – a 12-month multicenter cohort study from the German early access program
Source: eClinicalMedicine. 2024 Feb 15;69:102495. doi: 10.1016/j.eclinm.2024.102495 (PMC10878861; doi:10.1016/j.eclinm.2024.102495)
Supplement: Supplementary Table S2 [file mmc2.docx]

**Supplementary table 2: Demographic, clinical, and laboratory data related to ALSFRS-R progression rate (responders vs. non-responders) during tofersen treatment**

P-values were calculated with unpaired Student’s t-test for continuous variables, Chi-Square-test for nominal variables and non-parametric Mann-Whitney U test for non-normally distributed variables. A p-value of ≤ 0·05 was regarded as statistically significant.

ALSFRS-R: ALS Functional Rating Scale-Revised, CSF: cerebrospinal fluid, NfL: neurofilament light chain, pNfH: phosphorylated neurofilament heavy chain, *_1_NP_000445.1, _2_NM_000454.5*

|  | **Slower ALSFRS-R progression rate during tofersen treatment compared to pre-baseline (*n* = 17)** | **Faster ALSFRS-R progression rate during tofersen treatment compared to pre-baseline (*n* = 6)** | **P-value** |
| --- | --- | --- | --- |
| **Observation period** (months) (median, IQR) | 6·0 (2·8-11·5) (*n* = 17) | 3·5 (2·6-8·9) (*n* = 6) | 0.55 |
| **Age** (years) (median, IQR) | 54·0 (43·0-63·5) (*n* = 17) | 53·0 (35·5-58·8) (*n* = 6) | 0.40 |
| **Sex** |  |  | 0.41 |
| male | 47·1 % (*n* = 8) | 66·7 % (*n* = 4) |  |
| female | 52·9 % (*n* = 9) | 33·3 % (*n* = 2) |  |
| **Onset** |  |  | 0.54 |
| spinal | 94·1 % (*n* = 16) | 100·0 % (*n* = 6) |  |
| bulbar | 5·9 % (*n* = 1) | 0·0 % (*n* = 0) |  |
| **Type** |  |  | 0.90 |
| sporadic | 47·1 % (*n* = 8) | 50·0 % (*n* = 3) |  |
| familial | 52·9 % (*n* = 9) | 50·0 % (*n* = 3) |  |
| **ALSFRS-R** (baseline) (median, IQR) | 35·0 (28·0-41·5) (*n* = 17) | 39·5 (36·5-44·5) (*n* = 6) | 0.20 |
| **ALSFRS-R** (last administration) (median, IQR) | 35·0 (29·5-42·0) (*n* = 17) | 36·0 (26·0-43·0) (*n* = 6) | 0.96 |
| **Progression Rate pre-baseline** (ALSFRS-R points lost/month; median, IQR) | 0·41 (0·20-0·77) (*n* = 17) | 0·26 (0·10-1.06) (*n* = 6) | 0.65 |
| **Progression Rate during tofersen treatment** (ALSFRS-R points lost/month; median, IQR) | 0·00 (-0·31-0·18) (*n* = 17) | 0·98 (0·32-1.81) (*n* = 6) | **<0.001** |
| **Disease duration** (median, IQR) | 29·5 (15·1-58·3) months (*n* = 17) | 15·6 (7·7-89·2) months (*n* = 6) | 0.43 |
| **NfL in serum at baseline** (pg/ml) (median, IQR) | 70 (36-145) (*n* = 17) | 129 (35-271) (*n* = 6) | 0.40 |
| **pNfH in CSF at baseline** (pg/ml) (median, IQR) | 1748 (970-4937) (*n* = 12) | 4703 (1352-9696) (*n* = 6) | 0.34 |
| **NfL in serum at last administration** (pg/ml) (median, IQR) | 29 (20-59) (*n* = 17) | 71 (28-109) (*n* = 6) | 0.22 |
| **pNfH in CSF at last administration** (pg/ml) (median, IQR) | 987 (555-2133) (*n* = 12) | 1751 (455-2606) (*n* = 6) | 0.55 |
| ***SOD1* mutation spectrum** | p.Val15*Met_1_ (c.43G>A_2_), heterozygous  p.Gly42Asp_1_ (c.125G>A_2_), heterozygous  p.His44Arg_1_ (c.131A>G_2_), heterozygous  p.Val88Met_1_ (c.262G>A_2_), heterozygous  p.Asp91Ala_1_ (c.272A>C_2_), homozygous (*n*=3), heterozygous (*n*=1)  p.Ile114Thr_1_ (c.341T>C_2_), heterozygous  p.Arg116Gly_1_ (c.346C>G_2_), heterozygous (*n*=3)  p.Val119_Val12_1_ (c.358-10T>G_2_), heterozygous  p.Glu134*_1_ (c.396_399dup_2_), heterozygous  p.Leu145Phe_1_ (c.435G>T_2_), heterozygous (*n*=2)  p.Val149Ala_1_ (c.446T>C_2_), heterozygous | p.His47Arg (c.140A>G), heterozygous  p.Asp91Val (c.272A>T), heterozygous  p.Gly94Cys (c.280G>T), heterozygous  p.Arg116Gly (c.346C>G), heterozygous  p.Leu118Val (c.352C>G), heterozygous  p.Leu145Phe (c.435G>T), heterozygous |  |
