# Supplementary material for: Effects of tofersen treatment in patients with SOD1-ALS in a “real-world” setting – a 12-month multicenter cohort study from the German early access program
Source: eClinicalMedicine. 2024 Feb 15;69:102495. doi: 10.1016/j.eclinm.2024.102495 (PMC10878861; doi:10.1016/j.eclinm.2024.102495)
Supplement: Supplementary Table S3 [file mmc3.docx]

**Supplementary table 3. ALSFRS-R, progression rate and biomarkers related to tofersen treatment time.**

*Median treatment time in patients with > 6 months treatment with tofersen 11*·*0 months (IQR 8*·*0-12*·*0 months). Median treatment time in patients with < 6 months treatment with tofersen 2*·*8 months (IQR 1*·*9-3*·*7 months). Changes over time were analyzed by Wilcoxon matched-pairs signed rank test. A P-value of ≤ 0*·*05 was regarded as statistically significant. ALSFRS-R: Amyotrophic lateral sclerosis functional rating scale revised, NfL: neurofilament light chain, pNfH: phosphorylated neurofilament heavy chain.*

|  | **pre-baseline** | **at last administration** | **P-value** |
| --- | --- | --- | --- |
| **Patients > 6 months treatment with tofersen (*n* = 12)** | | | |
| **ALSFRS-R** (median, IQR) | 33·0 (29·8-41·8) (*n* = 12) | 31·5 (23·5-38·0) (*n* = 12) | 0.40 |
| **Progression Rate** (ALSFRS-R points lost/month; median, IQR) | 0·43 (0·16-0·88) (*n* = 12) | 0·06 (-0.06-0.30) (*n* = 12) | 0.05 |
| **NfL in serum** (pg/ml) (median, IQR) | 80 (36-170) (*n* = 12) | 28 (18-75) (*n* = 12) | **0.002** |
| **pNfH in CSF** (pg/ml) (median, IQR) | 1748 (932-6450) (*n* = 10) | 863 (521-2891) (*n* = 10) | **0.01** |
|  | | | |
| **Patients < 6 months treatment with tofersen (*n* = 12)** | | | |
| **ALSFRS-R** (median, IQR) | 41·0 (36·0-44·0) (*n* = 11) | 40·0 (35·0-43·0) (*n* = 11) | 0.42 |
| **Progression Rate** (ALSFRS-R points lost/month; median, IQR) | 0·28 (0·20-0·52) (*n* = 11) | 0·20 (-0·36-0·36) (*n* = 11) | 0.32 |
| **NfL in serum** (pg/ml) (median, IQR) | 70 (41-144) (*n* = 11) | 47 (26-64) (*n* = 11) | **0.004** |
| **pNfH in CSF** (pg/ml) (median, IQR) | 3422 (1110-7998) (*n* = 8) | 1446 (590-2197) (*n* = 8) | **0.04** |
