# Supplementary material for: Effects of tofersen treatment in patients with SOD1-ALS in a “real-world” setting – a 12-month multicenter cohort study from the German early access program
Source: eClinicalMedicine. 2024 Feb 15;69:102495. doi: 10.1016/j.eclinm.2024.102495 (PMC10878861; doi:10.1016/j.eclinm.2024.102495)
Supplement: Supplementary Table S4 [file mmc4.docx]

**Supplementary table 4: Laboratory findings in CSF and serum**

| **Maximum leukocyte count (cells/µl)** (median, IQR) | 15·0 (4·7-45·0) (*n* = 15) |
| --- | --- |
| **Pleocytosis in CSF (leukocytes ≥ 5/µl)** | 73·3 % (*n* = 11/15) |
| **First occurrence of pleocytosis (weeks)** (median, IQR) | 3·7 (1·9–14·2) (*n* = 9) |
| **Maximum protein level (mg/l)** (median, IQR) | 610 (479-852) (*n* = 17) |
| **First occurrence of elevated protein levels during tofersen therapy** | 47·1 % (*n* = 8/17) |
| **First occurrence of elevated protein levels (weeks)** (median, IQR) | 20·4 (5·5-44·7) (*n* = 8) |
| **Maximum lactate (mmol/l)** (median, IQR) | 1·87 (1·66-2·22) (*n* = 15) |
| **First occurrence of intrathecal immunoglobulin synthesis** | IgM 90 % (*n* = 9/10) |
| **(IgM, IgA, IgG) during tofersen therapy** | IgA 30 % (*n* = 3/10) |
| (percentage of patients with available data) | IgG 20 % (*n* = 2/10) |
| **First occurrence of oligoclonal bands (OCB)**  (percentage of patients with available data; OCB pre-existing *n* = 6) | 10 % (*n* = 1/10) |
