# Supplementary material for: Effects of tofersen treatment in patients with SOD1-ALS in a “real-world” setting – a 12-month multicenter cohort study from the German early access program
Source: eClinicalMedicine. 2024 Feb 15;69:102495. doi: 10.1016/j.eclinm.2024.102495 (PMC10878861; doi:10.1016/j.eclinm.2024.102495)
Supplement: Worksheet Tofersen [file mmc5.pdf]

Tofersen Minimal Data Set MND-NET

MND-NET German Network for Motor Neuron Diseases

Patient:________________________________
Date of Birth:__________________________
Date:___________________________________

1. Pre-Dose Vital Signs

Vital Signs – Pre-Dose
Time collected: _____:_____
BP: _________/__________mmHg HR: __________ bpm RR: _________ breaths/minute
O2 Sat: ______________ % ☐ Room Air ☐ On BiPAP

2. Pre-Dose Slow Vital Capacity

Slow Vital Capacity ☐ Not Done				
Was facemask used? ____________				
Predicted SVC Result (Liters): ___________ Best SVC Result (%): __________				
**Trial 1**	**Trial 2**	**Trial 3**	**Trial 4**	**Trial 5**
_____________ liters	_____________ liters	_____________ liters	_____________ liters	_____________ liters

3. ECG Finding

12 lead ECG
**ECG Finding:**____

4. ALSFRS-R

ALSFRS-R (appendix 1)
**Score:**___________

5. ALSFRS-R (self-rated via ALS App)

ALSFRS-R (self-rated via ALS App)
Score: _______

6. Neurological Examination: Baseline and every 3 months
appendix 2

7. Motorscale: Baseline and every 3 months
appendix 9

8. Quality of Life (EQ-5D-5L): Baseline and every 3 months
appendix 3

9. Safety Laboratory and Biosampling
→ appendix 4 **SOP Biosampling,** → appendix 5 **Filling Instruction,** → appendix 6 **Material Transfer**

Safety Laboratory and Biosampling

Safety Laboratory Blood:

☐ Blood Count	☐ Coagulation
☐ Transaminases	☐ Creatinine
☐ CRP	

Safety Laboraty CSF:

☐ Cell Count	☐ Glucose
☐ Total Protein	☐ Lactate
☐ Albumin Quotient	☐ CSF Pressure

Biosampling MND-NET:
appendix 6

☐ Serum 7,5ml	☐ CSF	☐ EDTA 7,5ml

10. Lumbar Puncture and Drug Administration

**Lumbar Puncture**
**Needle in time: ___:___ Needle out time: ___:___ # of Attempts: _____**
**Interspace(s):☐L2/3 ☐ L3/4 ☐ L5/S1**
**Needle used for collection: ________ G ☐ Atraumatic ☐ Traumatic**
Sample volume ______ ml
**LP Procedure done by: ____ (Name, Signature & Date)**
Additional notes: ____

**Drug Administration**	
**Start time**	____:____
Stop time	____:____
Dose	
Volume administered	
Route	Intrathecal
**Administered by:___________________Signature:_________________ Date:________________**	
Additional notes: ____	

11. Post-Dose Vital Signs

**Vital Signs – Post-Dose – 60 min post dose**
Time collected: ____:____   BP: ________/_________mmHg HR: _________ bpm RR: ________ breaths/minute   O2 Sat: _____________ % ☐ Room Air ☐ On BiPAP

12. Safety Follow up call 24 h after LP:__________________

Overview Appendices

Appendix 1 ALSFRS-R	pages 7 - 11
Appendix 2 Neurological Examination	pages 15
Appendix 3 EQ-5D	pages 12 - 13
Appendix 4 SOP Biosampling	page 22
Appendix 5 Filling Instruction	page 23
Appendix 6 Material Transfer	page 24
Appendix 7 Co-Medication	page 5
Appendix 8 Adverse Events	page 6
Appendix 9 Motorscale	page 14

Back to Top

Appendix 7 Co-Medication

Nr.	Medication	Start Date / Time	Stop Date / Time	Indication	Route	Dose/Units	Frequency	ongoing

Back to Overview
Back to Top

Appendix 8 Adverse Events

Nr.	**Adverse Event**	**Start Date/time**	**Stop Date/time**	**Severity**	**Outcome**	**Related to study drug**	**Related to LP**	**Action taken with study drug**	**serious (y/n)**	**CoMed Y/N**	**Signature**

[Back to Overview](#)
[Back to Top](#)

Appendix 1 ALSFRS-R (German Consent Version)

ALSFRS-R **konsentierte Version**

ALSFRS-R - Skala zur Beurteilung der Körperfunktionen bei Amyotropher Lateralsklerose (ALS)
(ALSFRS-R, Amyotrophic Lateral Sclerosis Functional Rating Scale – Revised)

Die ALS-Skala, ALSFRS-R, umfasst motorische Funktionsbereiche, die typischerweise durch die ALS eingeschränkt sein können. Die Bewertung kann sowohl von einer anderen Person (z.B. betreuende/r Arzt/Ärztin, Angehörige/r, Pflegepersonal) nach Befragung der an ALS erkrankten Person als auch von dieser selbst durchgeführt werden. Bitte lesen Sie sowohl die Antworten als auch die Erläuterungen sorgfältig durch und bewerten Sie die Funktion bzw. deren ALS-bedingte Einschränkung nach der zum aktuellen Zeitpunkt bestehenden Fähigkeit.

Wenn in einem Funktionsbereich eine Einschränkung vorliegt, die entweder eine andere medizinische Ursache als die ALS hat oder die bereits vor Beginn der ALS bestand (z.B. Einschränkung des Laufens durch eine Hüft-Prothese), kann dieser Bereich als "nicht eingeschränkt" bewertet werden (4 Punkte). Die Funktion sollte immer im Vergleich zu der Zeit vor den ersten ALS-Symptomen beurteilt werden. Es kann von dieser Empfehlung abgewichen werden, wenn Einschränkungen hinzugetreten sind, die wahrscheinlich auf die ALS zurückzuführen sind. Diese Vorgehensweise sollte bei allen Punkten und in zukünftigen ALSFRS-R-Bewertung eingehalten werden.

1. Sprechen
Bitte wählen Sie eine der folgenden Antworten:

- (4) ☐ Ungestört
  Das Sprechen bzw. die Artikulation ist genauso wie vor dem Auftreten der ersten ALS-Symptome.
- (3) ☐ Wahrnehmbare Sprechstörungen
  Eine neu aufgetretene Veränderung beim Sprechen, der Artikulation oder der Stimmgebung (Phonation), z. B. im Sinne verwaschenen Sprechens oder einer Heiserkeit, wird entweder selbst oder vom näheren Umfeld bemerkt.
- (2) ☐ Wiederholung zur Verständigung notwendig
  Es bedarf öfters einer Wiederholung einzelner Worte oder Satzteile, um sich verständlich zu machen.
- (1) ☐ Das Sprechen wird mit nonverbaler Kommunikation kombiniert
  Schreiben, Verwendung von Kommunikationshilfen oder ähnliche Methoden werden eingesetzt, um das Sprechen zu ergänzen.
- (0) ☐ Verlust des verständlichen Sprechens
  Der Einsatz von Kommunikationshilfen oder ähnlichen Methoden ist durchgehend notwendig.

2. Speichelfluss
Bitte wählen Sie eine der folgenden Antworten:

- (4) ☐ Ungestört
  Es sammelt sich kein vermehrter Speichel im Mund.
- (3) ☐ Geringfügig, aber eindeutiges Übermaß an Speichel im Mund; nachts Speichelverlust möglich
  Vermehrte Speichelansammlung im Mund, jedoch ohne subjektive Beeinträchtigung und ohne Speichelverlust am Tage.
- (2) ☐ Mäßig vermehrter Speichelfluss; geringer Speichelverlust möglich
  Gelegentlich wird ein Taschentuch verwendet, um tagsüber die Mundwinkel abzutrocknen.
- (1) ☐ Deutlich vermehrter Speichelfluss mit etwas Speichelverlust
  Speichelverlust ist regelmäßig vorhanden, und ein Taschentuch wird oft, aber nicht immer, verwendet.
- (0) ☐ Ausgeprägter Speichelverlust aus dem Mund
  Die ständige Verwendung eines Taschentuchs oder einer Absaugung ist erforderlich.

1

Version 2.0, letztes Update 17.01.2022

Back to Overview

Back to Top

ALSFRS-R **konsentierte Version**

3. Schlucken
Bitte wählen Sie eine der folgenden Antworten:

[4] ☐ Ungestört
Das Schlucken jeglicher Nahrung und von Flüssigkeiten erfolgt ohne Probleme.

[3] ☐ Beginnende Essprobleme mit gelegentlichem Verschlucken
Es wird mehr Zeit zur Nahrungsaufnahme benötigt, oder es werden kleinere Bissen zu sich genommen und mit Vorsicht geschluckt. Es kommt gelegentlich zum Verschlucken oder zu vermehrtem Husten.

[2] ☐ Änderung der Nahrungskonsistenz erforderlich
Die Beschaffenheit von Speisen und Getränken wird verändert oder Schwierigkeiten beim Schlucken führen zur Vermeidung bestimmter Konsistenzen (z. B. Fleisch, trockene Kekse, Nüsse). Ggf. werden wegen einer Schluckstörung ergänzend Zusatznahrung oder Andickungsmittel verwendet.

[1] ☐ Ergänzende Sondenernährung erforderlich
Die Nahrungsaufnahme ist aufgrund der Schluckstörung so schwierig, dass eine Ernährungssonde (PEG) bereits angelegt oder aus ärztlicher Sicht dringend empfohlen wurde, um die Kalorienzufuhr zu ergänzen bzw. die Gefahr des Verschluckens zu vermindern.

[0] ☐ Ernährung ausschließlich über eine Ernährungssonde
Die Aufnahme von Nahrung und Flüssigkeit erfolgt ausschließlich über eine Ernährungssonde. Die orale Nahrungsaufnahme ist aufgrund einer hochgradigen Schluckstörung unmöglich.

4. Handschrift
Bewertet wird das Schreiben mit der Schreibhand in üblicher Haltung.
Bitte wählen Sie eine der folgenden Antworten:

[4] ☐ Ungestört
Das Schreiben mit der üblich verwendeten Schreibhand ist ohne Probleme möglich.

[3] ☐ Langsamer oder unsauber, alle Wörter sind lesbar
Das Schreiben gestaltet sich schwierig oder die Schrift hat sich verändert, selbst wenn die geschriebenen Wörter gut lesbar sind.

[2] ☐ Nicht alle Wörter sind lesbar
Im geschriebenen Text sind einzelne Wörter nicht lesbar. Um lesbar zu schreiben, werden Hilfsmittel wie Schreibhilfen verwendet.

[1] ☐ Das Halten des Stiftes ist möglich, aber nicht das lesbare Schreiben
Der Stift kann gehalten werden. Das Schreiben über den eigenen Namen oder die eigene Unterschrift hinaus ist jedoch nicht möglich.

[0] ☐ Kann Stift nicht halten
Das Halten eines Stiftes, z. B. zum Unterschreiben, ist nicht möglich.

5.a Essen schneiden und Gebrauch von Besteck
Für Personen, die nicht regelhaft eine Ernährungssonde zur Kalorienzufuhr nutzen.
Bitte wählen Sie eine der folgenden Antworten:

[4] ☐ Ungestört
Die Benutzung von Besteck ist ohne Probleme möglich (als Probleme gelten z. B. Messer und Gabel statt Essstäbchen oder die Neigung, vermehrt einen Löffel zu benutzen).

[3] ☐ Etwas langsam und unbeholfen, aber keine Hilfe erforderlich
Aufgrund der Beeinträchtigung der Hände wird mehr Zeit zum Essen benötigt. Die Benutzung von Besteck ist verändert, jedoch selbständig möglich.

[2] ☐ Kann die meisten Speisen schneiden, aber langsam und unbeholfen, braucht teilweise Hilfe
Beim Schneiden bestimmter Speisen wird gelegentlich die Hilfe von einer anderen Person benötigt, oder es werden Hilfsmittel, z. B. in Form von speziellem Besteck, verwendet.

[1] ☐ Essen muss geschnitten werden, kann langsam alleine essen
Für das Schneiden von festen Lebensmitteln ist meistens oder immer eine Unterstützung erforderlich. Selbständiges Essen ist aber möglich (z. B. mit Gabel oder Löffel).

[0] ☐ Essen muss gereicht werden
Nahrung wird immer angereicht. Die selbständige Benutzung von Besteck (z. B. Gabel oder Löffel) ist nicht möglich.

2

Version 2.0, letztes Update 17.01.2022

Back to Overview

Back to Top

ALSFRS-R **konsentierte Version**

5.b Ernährungssonde und Zubehör handhaben
Für Personen, die regelhaft eine Ernährungssonde zur Kalorienzufuhr nutzen. Bewertet werden soll die vorhandene manuelle Fähigkeit.
Bitte wählen Sie eine der folgenden Antworten, wenn überwiegend eine Ernährungssonde zur Nahrungsaufnahme genutzt wird:

- [4] ☐ Ungestört
  Die Sonde kann problemlos selbst versorgt und Verschlüsse und Packungen können selbständig geöffnet oder geschlossen werden.
- [3] ☐ Etwas langsam und unbeholfen, aber alle Handgriffe können selbständig ausgeführt werden
  Es wird keine Unterstützung bei der Handhabung der Ernährungssonde benötigt, allerdings ist der Umgang erschwert.
- [2] ☐ Beim Umgang mit Verschlüssen und Deckeln teilweise Hilfe erforderlich
  Der Umgang mit der Sonde ist überwiegend selbständig möglich. Hilfe wird jedoch z. B. beim Öffnen von Verschlüssen benötigt.
- [1] ☐ Minimale Unterstützung kann bei der Sondenversorgung gegeben werden
  Die Versorgung der Ernährungssonde muss überwiegend von einer anderen Person übernommen werden. Minimale Handgriffe können selbständig getätigt werden.
- [0] ☐ Kann an keiner Stelle bei der Sondenversorgung mithelfen
  Die Versorgung der Ernährungssonde muss vollständig von einer anderen Person übernommen werden. Es können keine Handgriffe selbst getätigt werden.

6. Ankleiden und Körperpflege
Bitte wählen Sie eine der folgenden Antworten:

- [4] ☐ Ungestört
  Das Ankleiden und die Körperpflege sind ohne Probleme möglich.
- [3] ☐ Keine Hilfe erforderlich, jedoch mit Mühe verbunden
  Das Ankleiden oder die Körperpflege sind langsamer als zuvor, bleiben jedoch unabhängig und ohne Unterstützung durch Hilfsverfahren oder durch eine andere Person möglich.
- [2] ☐ Zeitweilig sind Hilfe oder Hilfsverfahren erforderlich
  Es wird gelegentlich Hilfe durch eine Person in Anspruch genommen, oder es werden Strategien entwickelt, um Beeinträchtigungen auszugleichen (z. B. leicht an- und ausziehbare Kleidung, Sitzen beim Ankleiden oder Duschen, Nutzung von Hilfsmitteln).
- [1] ☐ Hilfe durch andere Personen erforderlich
  Beim Ankleiden oder der Körperpflege ist regelmäßig die Unterstützung durch eine andere Person notwendig.
- [0] ☐ Vollständige Abhängigkeit
  Das Ankleiden und die Körperpflege müssen vollständig von einer anderen Person übernommen werden.

7. Umdrehen im Bett und Richten der Bettdecke
Bitte wählen Sie eine der folgenden Antworten:

- [4] ☐ Ungestört
  Das Umdrehen im Bett und Richten der Bettdecke lassen sich ohne Probleme durchführen.
- [3] ☐ Etwas langsam und unbeholfen, aber keine Hilfe erforderlich
  Es ist schwieriger, sich im Bett umzudrehen oder die Bettdecke zu richten.
- [2] ☐ Selbständig möglich, jedoch mit großer Mühe verbunden
  Das Umdrehen im Bett und das Richten der Bettdecke sind nur mit großer Mühe möglich. Eventuell wird eine der beiden Tätigkeiten unterstützt oder es wird zum Umdrehen ein Haltegriff verwendet.
- [1] ☐ Kann zum Umdrehen oder Richten der Bettdecke ansetzen, aber die Bewegung nicht selbständig vollenden
  Das Drehen im Bett oder Richten der Bettdecke kann begonnen werden. Es wird jedoch Unterstützung durch eine Person oder Hilfsmittel benötigt, um die jeweilige Tätigkeit zu vollenden.
- [0] ☐ Vollständige Abhängigkeit
  Beim Umdrehen im Bett und dem Richten der Bettdecke ist durchgehend Hilfe erforderlich

3

Version 2.0, letztes Update 17.01.2022

Back to Overview

Back to Top

ALSFRS-R konsentierte Version

8. Gehen
Bitte wählen Sie eine der folgenden Antworten:

[4] ☐ Ungestört
Es besteht keine Veränderung der Gehfähigkeit.

[3] ☐ Beginnende Gehschwierigkeiten
Es zeigen sich Veränderungen, die eine Verlangsamung, Unsicherheit oder Stolpern einschließen. Hilfe in Form einer anderen Person, durch Festhalten an Gegenständen oder durch die Verwendung von Gehhilfen außerhalb des Wohnbereichs (z. B. Fußheberschiene, Gehstock, Rollator) wird jedoch nicht regelhaft benötigt.

[2] ☐ Gehen nur mit Unterstützung möglich
Das Gehen erfordert regelhaft ein Festhalten oder - außerhalb des Wohnbereichs - Hilfe durch Fußheberschienen, Gehhilfen oder durch eine andere Person.

[1] ☐ Nicht gehfähig, aber gezielte Bewegungen der Beine möglich
Die Beine können gezielt bewegt werden. Das Stehen mit Unterstützung, z. B. zum Transfer, kann möglich sein. Auch mit Unterstützung anderer Personen oder Gehhilfen besteht keine Gehfähigkeit.

[0] ☐ Keine zielgerichtete Beinbewegung möglich
Die Beine können das Körpergewicht nicht tragen (z. B. zum Transfer), und es können keine zweckdienlichen Beinbewegungen z. B. zur Erleichterung der Pflege ausgeführt werden.

9. Treppen hinaufsteigen
Bitte wählen Sie eine der folgenden Antworten:

[4] ☐ Ungestört
Es besteht keine Veränderung beim Hinaufsteigen einer Treppe.

[3] ☐ Langsam
Das Hinaufsteigen einer Treppe erfolgt verlangsamt, ist aber ohne Ruhepausen oder Unsicherheit möglich.

[2] ☐ Leichte Unsicherheit oder Ermüdung
Das Hinaufsteigen einer Treppe ist unsicherer. Eventuell sind Ruhepausen nötig. Hilfe, z. B. durch einen Handlauf oder eine andere Person, ist nicht zwingend erforderlich.

[1] ☐ Unterstützung erforderlich
Das Hinaufsteigen einer Treppe ist ohne die Verwendung eines Handlaufs oder die Hilfe einer Person nicht möglich.

[0] ☐ Nicht möglich
Treppen können auch mit Hilfe nicht hinaufgestiegen werden.

10. Luftnot und Kurzatmigkeit
Bitte wählen Sie eine der folgenden Antworten:

[4] ☐ Ungestört
Luftnot oder Kurzatmigkeit tritt bei üblicher Belastung im Alltag nicht auf.

[3] ☐ Beim Gehen
Luftnot oder Kurzatmigkeit tritt beim Gehen in Schrittgeschwindigkeit oder bei mäßiger Belastung auf.

[2] ☐ Auftreten bei Aktivitäten des täglichen Lebens einschließlich Essen, Baden, Ankleiden
Luftnot oder Kurzatmigkeit tritt bei geringer Belastung oder längerem Sprechen auf.

[1] ☐ Auftreten in Ruhe, im Sitzen oder im Liegen
Luftnot oder Kurzatmigkeit besteht ohne Belastung entweder im Sitzen oder im Liegen oder in beiden Positionen.

[0] ☐ Erhebliche Luftnot oder Kurzatmigkeit in Ruhe. Eine mechanische Beatmung wird aufgrund der Luftnot oder einer Kurzatmigkeit genutzt oder empfohlen.
Luftnot oder Kurzatmigkeit ist in Ruhe immer deutlich vorhanden und/oder eine Maskenbeatmung (nicht-invasive Beatmung) oder Beatmung über Luftröhrenschnitt (Tracheostoma) muss zur Linderung von Luftnot und Kurzatmigkeit eingesetzt werden.

4

Version 2.0, letztes Update 17.01.2022

[Back to Overview](#)

[Back to Top](#)

ALSFRS-R konsentierte Version

11. Atmungsbedingte Schlafstörung
Bitte wählen Sie eine der folgenden Antworten:

[4] ☐ Ungestört
Das Ein- und Durchschlafen ist nicht durch Luftnot oder Kurzatmigkeit beeinträchtigt.

[3] ☐ Wiederholte Schlafstörung durch Luftnot oder Kurzatmigkeit, keine Verwendung von mehr als zwei Kissen
Es bestehen bereits beim Hinlegen oder während der Nacht Luftnot oder Kurzatmigkeit. Das Schlafen auf der Seite kann die Atmung verbessern. Der Oberkörper wird nicht durch mehr als zwei Kopfkissen oder mittels eines um mehr als 30 Grad hochgestellten Kopfteils aufgerichtet.

[2] ☐ Regelmäßige Verwendung von mehr als zwei Kissen zum Schlafen erforderlich
In flacher Rückenlage ist das Atmen merklich unangenehm und das Ein- oder Durchschlafen dadurch gestört. Der Oberkörper wird durch drei oder mehr Kissen oder mittels eines um mehr als 30 Grad hochgestellten Kopfteils aufgerichtet.

[1] ☐ Schlafen nur im Sitzen möglich
Zum Schlafen ist eine Sitzposition entweder im Bett oder in einem Stuhl notwendig.

[0] ☐ Hochgradige Schlafstörung aufgrund von Luftnot oder Kurzatmigkeit
Aufgrund von Luftnot oder Kurzatmigkeit ist das Schlafen ohne Maskenbeatmung (nicht-invasive Beatmung) oder Beatmung über Luftröhrenschnitt (Tracheostoma) nicht möglich. Eine mechanische Beatmung wird zur Beschwerdelinderung regelhaft genutzt.

12. Mechanische Beatmung
Bitte wählen Sie eine der folgenden Antworten:

[4] ☐ Nicht erforderlich
Die Atmung erfolgt immer selbständig ohne die Nutzung einer mechanischen Beatmung. Eine nächtliche Druckunterstützung (z. B. CPAP-Therapie zur Behandlung eines Schlafapnoe-Syndroms) zählt nicht als mechanische Beatmung.

[3] ☐ Zeitweilige Nutzung einer nicht-invasiven Beatmung
Eine Maskenbeatmung (nicht-invasive Beatmung, z. B. BiPAP) wird unregelmäßig oder für weniger als die Dauer des Nachtschlafs eingesetzt.

[2] ☐ Ständige Nutzung einer nicht-invasiven Beatmung in der Nacht oder zusätzlich stundenweise am Tag
Eine Maskenbeatmung (nicht-invasive Beatmung) wird regelmäßig während der Nacht oder zusätzlich stundenweise während des Tages eingesetzt (insgesamt 8 bis 22 Stunden täglich bezogen auf 24 Stunden).

[1] ☐ Kontinuierliche Nutzung einer nicht-invasiven Beatmung (Tag und Nacht)
Eine Maskenbeatmung (nicht-invasive Beatmung) wird nahezu die gesamt Zeit eingesetzt (mehr als 22 Stunden täglich bezogen auf 24 Stunden).

[0] ☐ Mechanische Beatmung über Intubation oder Luftröhrenschnitt (Tracheostoma)
Es erfolgt eine kontinuierliche mechanische Beatmung über einen Beatmungsschlauch (Intubation) oder einen Luftröhrenschnitt (Tracheostoma).

Back to Overview

Back to Top

Appendix 3 EQ-5D-5L German Version (Baseline and every 3 months)

Bitte kreuzen Sie unter jeder Überschrift DAS Kästchen an, das Ihre Gesundheit HEUTE am besten beschreibt.

BEWEGLICHKEIT / MOBILITÄT

Ich habe keine Probleme, herumzugehen ❑
Ich habe leichte Probleme, herumzugehen ❑
Ich habe mäßige Probleme, herumzugehen ❑
Ich habe große Probleme, herumzugehen ❑
Ich bin nicht in der Lage, herumzugehen ❑

FÜR SICH SELBST SORGEN

Ich habe keine Probleme, mich selbst zu waschen oder anzuziehen ❑
Ich habe leichte Probleme, mich selbst zu waschen oder anzuziehen ❑
Ich habe mäßige Probleme, mich selbst zu waschen oder anzuziehen ❑
Ich habe große Probleme, mich selbst zu waschen oder anzuziehen ❑
Ich bin nicht in der Lage, mich selbst zu waschen oder anzuziehen ❑

ALLTÄGLICHE TÄTIGKEITEN ***(z. B. Arbeit, Studium, Hausarbeit, Familien- oder Freizeitaktivitäten)***

Ich habe keine Probleme, meinen alltäglichen Tätigkeiten nachzugehen ❑
Ich habe leichte Probleme, meinen alltäglichen Tätigkeiten nachzugehen ❑
Ich habe mäßige Probleme, meinen alltäglichen Tätigkeiten nachzugehen ❑
Ich habe große Probleme, meinen alltäglichen Tätigkeiten nachzugehen ❑
Ich bin nicht in der Lage, meinen alltäglichen Tätigkeiten nachzugehen ❑

SCHMERZEN / KÖRPERLICHE BESCHWERDEN

Ich habe keine Schmerzen oder Beschwerden ❑
Ich habe leichte Schmerzen oder Beschwerden ❑
Ich habe mäßige Schmerzen oder Beschwerden ❑
Ich habe starke Schmerzen oder Beschwerden ❑
Ich habe extreme Schmerzen oder Beschwerden ❑

ANGST / NIEDERGESCHLAGENHEIT

Ich bin nicht ängstlich oder deprimiert ❑
Ich bin ein wenig ängstlich oder deprimiert ❑
Ich bin mäßig ängstlich oder deprimiert ❑
Ich bin sehr ängstlich oder deprimiert ❑
Ich bin extrem ängstlich oder deprimiert ❑

2

Germany (German) v.2 © 2010 EuroQol Group. EQ-5D™ is a trade mark of the EuroQol Group

Back to Overview

Back to Top

- Wir wollen herausfinden, wie gut oder schlecht Ihre Gesundheit HEUTE ist.
- Diese Skala ist mit Zahlen von 0 bis 100 versehen.
- 100 ist die <u>beste</u> Gesundheit, die Sie sich vorstellen können.
  0 (Null) ist die <u>schlechteste</u> Gesundheit, die Sie sich vorstellen können.
- Bitte kreuzen Sie den Punkt auf der Skala an, der Ihre Gesundheit HEUTE am besten beschreibt.
- Jetzt tragen Sie bitte die Zahl, die Sie auf der Skala angekreuzt haben, in das Kästchen unten ein.

IHRE GESUNDHEIT HEUTE = ☐

Beste Gesundheit, die Sie sich vorstellen können

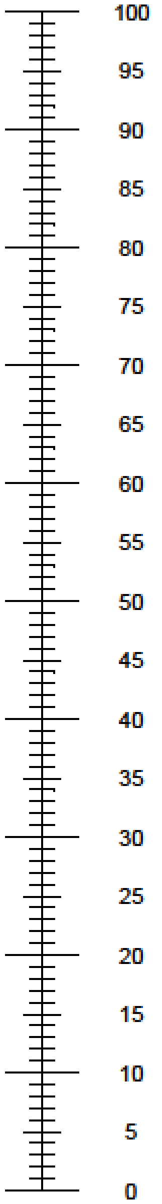

Schlechteste Gesundheit, die Sie sich vorstelle können

3

Germany (German) v.2 © 2010 EuroQol Group. EQ-5D™ is a trade mark of the EuroQol Group

Back to Overview

Back to Top

Appendix 9 Motorscale (Baseline and every 3 months)

**Kraftgrade rechts**	0/5	1/5	2/5	3/5	4/5	5/5
Armabduktion						
Armflexion						
Handstreckung						
Fingerstreckung						
Fingerspreizung						
Daumenabduktion						
Hüftbeugung						
Kniestreckung						
Kniebeugung						
Fußhebung						

**Kraftgrade links**	0/5	1/5	2/5	3/5	4/5	5/5
Armabduktion						
Armflexion						
Handstreckung						
Fingerstreckung						
Fingerspreizung						
Daumenabduktion						
Hüftbeugung						
Kniestreckung						
Kniebeugung						
Fußhebung						

Back to Overview

Back to Top

Appendix 2 Neurological Examination (Baseline and every 3 months)

Date of Examination:____________ Time of Examination:___________

Cranial Nerves:

	Status:	*Relationship:*	*Describe:*
Vision (II):	□ normal<br>□ abnormal<br>□ Not done	□ secondary to ALS<br>□ Other	____________
Eye Movements:<br>(III, IV, VI):	□ normal<br>□ abnormal<br>□ not done	□ secondary to ALS<br>□ Other	____________
Jaw movement and<br>Facial sensation (V):	□ normal<br>□ abnormal<br>□ not done	□ secondary to ALS<br>□ Other	____________
Facial motion (VII):	□ normal<br>□ abnormal<br>□ not done	□ secondary to ALS<br>□ Other	____________
Hearing (VIII):	□ normal<br>□ abnormal<br>□ not done	□ secondary to ALS<br>□ other	____________
Swallowing, pharynx,<br>Larynx (IX, X):	□ normal<br>□ abnormal<br>□ not done	□ secondary to ALS<br>□ other	____________

Back to Overview

Back to Top

Status:	Relationship:	Describe:	
SCM, Trapezius (XI):	□ normal	□ secondary to ALS	____________
	□ abnormal	□ other	
	□ not done		
Tongue (XII):	□ normal	□ secondary to ALS	____________
	□ abnormal	□ other	
	□ other		

Coordination/Cerebellar Function:

	Status:	Relationship:	Describe:
Gait:	□ normal	□ secondary to ALS	____________
	□ abnormal	□ other	
	□ not done		
Finger-Nose:	□ normal	□ secondary to ALS	____________
	□ abnormal	□ other	
	□ not done		
Heel-Shin:	□ normal	□ secondary to ALS	____________
	□ abnormal	□ other	
	□ not done		
Nystagmus:	□ not done	□ secondary to ALS	____________
	□ Absent	□ other	
	□ Present		
Tremor:	□ not done	□ secondary to ALS	____________
	□ Absent	□ other	
	□ Present		

Back to Overview

Back to Top

Rapid Rhythmic Movement:	□ not done	□ secondary to ALS	____________
	□ Absent	□ other	
	□ Present		

<u>Sensation Upper Extremities:</u>

	Status:	*Side:*	*Realtionship:*	*Describe:*
Pain/	□ not done	□ right	□ secondary to ALS	_____________
Temperature:	□ normal	□ left	□ other	
	□ abnormal	□ both		
Light Tough:	□ not done	□ right	□ secondary to ALS	_____________
	□ normal	□ left	□ other	
	□ abnormal	□ both		
Position:	□ not done	□ right	□ secondary to ALS	_____________
	□ normal	□ left	□ other	
	□ abnormal	□ both		
Vibration:	□ not done	□ right	□ secondary to ALS	_____________
	□ normal	□ left	□ other	
	□ abnormal	□ both		

<u>Sensation Lower Extremities:</u>

	Status:	*Side:*	*Realtionship:*	*Describe:*
Pain/	□ not done	□ right	□ secondary to ALS	_____________
Temperature:	□ normal	□ left	□ other	
	□ abnormal	□ both		
Light Tough:	□ not done	□ right	□ secondary to ALS	_____________
	□ normal	□ left	□ other	
	□ abnormal	□ both		
Position:	□ not done	□ right	□ secondary to ALS	_____________
	□ normal	□ left	□ other	
	□ abnormal	□ both		

Back to Overview

Back to Top

Vibration:	□ not done	□ right	□ secondary to ALS	____________
	□ normal	□ left	□ other	
	□ abnormal	□ both		

Neurological Examination Reflexes:

Upper Extremities:

	Status:	*Retained in wasted limb:*	*Relationship:*
Biceps right:	□ 0=absent	□ Yes	□ secondary to ALS
	□ 1=Trace	□ No	□ Other___________
	□ 2=normal	□ NA	
	□ 3=Brisk		
	□ 4=Clonus		
	□ unable to assess (e.g. amputation)		
	□ not done		
Biceps left:	□ 0= absent	□ Yes	□ secondary to ALS
	□ 1=Trace	□ No	□ Other___________
	□ 2=normal	□ NA	
	□ 3=Brisk		
	□ 4=Clonus		
	□ unable to assess (e.g. amputation)		
	□ not done		
Triceps right:	□ 0=absend	□ Yes	□ secondary to ALS
	□ 1=Trace	□ No	□ Other___________
	□ 2=normal	□ NA	
	□ 3=Brisk		
	□ 4=Clonus		
	□ unable to assess (e.g. amputation)		
	□ not done		

Back to Overview

Back to Top

Triceps left:	□ 0=absent	□ Yes	□ secondary to ALS
	□ 1=Trace	□ No	□ Other___________
	□ 2=normal	□ NA	
	□ 3=Brisk		
	□ 4=Clonus		
	□ unable to assess (e.g. amputation)		
	□ not done		

Lower Extremities:

	Status:	*Retained in wasted limb:*	*Relationship:*
Knee jerk	□ 0=absent	□ Yes	□ secondary to ALS
Right:	□ 1=trace	□ No	□ other ___________
	□ 2=normal	□ NA	
	□ 3=Brisk		
	□ 4=Clonus		
	□ unable to assess (e.g. amputation)		
	□ not done		
Knee jerk	□ 0=absent	□ Yes	□ secondary to ALS
Left:	□ 1=trace	□ No	□ other ___________
	□ 2=normal	□ NA	
	□ 3=Brisk		
	□ 4=Clonus		
	□ unable to assess (e.g. amputation)		
	□ not done		
Ankle jerk	□ 0=absent	□ Yes	□ secondary to ALS
Right:	□ 1=trace	□ No	□ other ___________
	□ 2=normal	□ NA	
	□ 3=Brisk		
	□ 4=Clonus		
	□ unable to assess (e.g. amputation)		
	□ not done		

Back to Top

Back to Overview

Ankle jerk	□ 0=absent	□ Yes	□ secondary to ALS
left:	□ 1=trace	□ No	□ other ___________
	□ 2=normal	□ NA	
	□ 3=Brisk		
	□ 4=Clonus		
	□ unable to assess (e.g. amputation)		
	□ not done		

Other:

	Status:	*Relationship:*
Jaw Jerk:	□ Not Done	□ secondary to ALS
	□ Absent	□ Other_________________
	□ Present	
	□ Pathologically Present	
Hoffman Sign right:	□ Not Done	□ secondary to ALS
	□ Absent	□ Other_________________
	□ Present	
	□ Pathologically Present	
	□ unable to assess (e.g. amputation)	
Hoffman Sign left:	□ Not Done	□ secondary to ALS
	□ Absent	□ Other_________________
	□ Present	
	□ Pathologically Present	
	□ unable to assess (e.g. amputation)	
Babinski right:	□ Not Done	□ secondary to ALS
	□ Absent	□ Other_________________
	□ Present	
	□ Pathologically Present	
	□ unable to assess (e.g. amputation)	

Back to Top

Back to Overview

Babinski left:

□ Not Done

□ Absent

□ Present

□ Pathologically Present

□ unable to assess (e.g. amputation)

□ secondary to ALS

□ Other__________________

Name________________________ Signature_______________________________ Date______________

Back to Overview

Back to Top

Appendix 4 SOP Biosampling

Protokoll für die Bioprobensammlung im MND-NET bei Behandlung mit Tofersen

I Bioproben zur Einlagerung in der Matrixbox - Bei jeder Tofersen-Applikation

Liquor:

1. Entnahmedatum und Entnahmezeit dokumentieren.
2. Liquor innerhalb von 15 min abzentrifugieren (1600g, RT, 10 min)
3. 3 ml Liquor in 12 x 250µl Aliquots abpipettieren und in die Matrix-Box stellen.
   In Appendix 8 Filling Instruction dokumentieren.
4. Matrixbox bei -80°C einfrieren. Die Zeit von Liquorentnahme bis zum Einfrosten soll 30 min nicht überschreiten

Serum 7,5 ml Monovette:

1. Serum 10 min nach Entnahme weiter bearbeiten (Serum benötigt etwas Zeit zum Gerinnen)
2. Für 10 min bei 2000g, 4°C zentrifugieren
3. Serum in 12 x 250 µl Aliquots abpipettieren und in die Matrixbox stellen.
   In Appendix 8 filling instruction dokumentieren.
4. Matrixbox bei -80°C einfrieren.

EDTA 7,5 ml Movovette:

1. Monovette mit Pseudonym und Tofersen-Gabe-Nummer labeln und ohne Weiterbearbeitung bei -80°C einfrieren.

II Bioproben mit sofortigem Versand zur Bestimmung der Neurofilamente

Die Bestimmung der Neurofilamente erfolgt an den Zeitpunkten:

Baseline, Applikation 1, Applikation 3, Applikation 6, Applikation 9 und Applikation 12

Vorgehen:

1. 1 ml Liquor in ein Eppendorf-Cup aliquotieren
2. 1 x 7,5 ml Serummonovette
3. Kühlakku für den Versand
4. Formular Material Transfer beilegen
5. Adresse:

Universitätsklinikum Ulm
Klinik für Neurologie
Neurobiobank
Oberer Eselsberg 45
89081 Ulm

SOP Biosampling Tofersen, 10.03.2022, V1.2

Back to Overview

Back to Top

Appendix 5 Filling Instruction

Befüllungstabelle Box Nummer __________

Pseudonym MND-NET	Material CSF, Serum	Position in der Box	Visite Nummer	Tofersen Gabe Nummer	Datum
		A1-A12			
		B1-B12			
		C1-C12			
		D1-D12			
		E1-E12			
		F1-D12			
		G1-E12			
		H1-H12			

Befüllungslayout der Matrixboxen

Box	1	2	3	4	5	6	7	8	9	10	11	12
A												
B												
C												
D												
E												
F												
G												
H												

250µl Probe pro Aliquot. Falls das Material nicht für 12 x 250µl ausreicht, die restlichen Plätze der entsprechenden Reihe leer lassen.

Filling Instruction Biosampling Tofersen, 17.02.2022, V1.0

Back to Overview

Back to Top

Appendix 6 Material Transfer

PROBENVERSAND
Neurobiobank Ulm

Projekt: MND-Netzwerk

➔ **Sofortiger Versand (ungekühlt)**

Pseudonym: ☐☐☐-☐☐☐-☐☐☐

Studienzentrum: ____________________

Visite:
- ☐ Baseline
- ☐ FUP 1
- ☐ FUP 2
- ☐ FUP 3
- ☐ FUP 4
- ☐ FUP 5
- ☐ FUP 6
- ☐ ________

Tofersen-Gabe-Nr. (0,1,2,...)________

Entnahmedatum: ____________________

Uhrzeit der Entnahme: ____________________

Probenversand am: ____________________

Uhrzeit Versand: ____________________

Beigefügt wurde:
- ☐ 1 x 7,5 ml Serum
- ☐ 1 x 1 ml Liquor
- ☐ __________

Lagerung bis zum Versand:
- Die Proben können im Kühlschrank über Nacht aufbewahrt werden
- Die Proben bitte nicht einfrieren

Wenn Liquor versendet wird, unbedingt mit Kühlakku versenden.

Abweichungen bitte hier dokumentieren:

____

____

Material Transfer 16.02.2022; V1.0

Back to Overview

Back to Top
